# Pro- and eukaryotic keystone taxa as potential bio-indicators for the water quality of subtropical Lake Dongqian

**DOI:** 10.3389/fmicb.2023.1151768

**Published:** 2023-04-26

**Authors:** Weihong Huang, Shuantong Li, Saisai Li, Hendrikus J. Laanbroek, Qiufang Zhang

**Affiliations:** ^1^College of Oceanology and Food Science, Quanzhou Normal University, Quanzhou, China; ^2^Zhejiang Wanli University, Ningbo, China; ^3^Department of Microbial Ecology, Netherlands Institute of Ecology (NIOO-KNAW), Wageningen, Netherlands; ^4^Ecology and Biodiversity Group, Department of Biology, Utrecht University, Utrecht, Netherlands

**Keywords:** bio-indicators, Lake Dongqian, microbial keystone taxa, 16S rRNA gene, 18S rRNA gene, spatial–temporal distribution, water properties

## Abstract

The microbial community plays an important role in the biogeochemical cycles in water aquatic ecosystems, and it is regulated by environmental variables. However, the relationships between microbial keystone taxa and water variables, which play a pivotal role in aquatic ecosystems, has not been clarified in detail. We analyzed the seasonal variation in microbial communities and co-occurrence network in the representative areas taking Lake Dongqian as an example. Both pro- and eukaryotic community compositions were more affected by seasons than by sites, and the prokaryotes were more strongly impacted by seasons than the eukaryotes. Total nitrogen, pH, temperature, chemical oxygen demand, dissolved oxygen and chlorophyll *a* significantly affected the prokaryotic community, while the eukaryotic community was significantly influenced by total nitrogen, ammonia, pH, temperature and dissolved oxygen. The eukaryotic network was more complex than that of prokaryotes, whereas the number of eukaryotic keystone taxa was less than that of prokaryotes. The prokaryotic keystone taxa belonged mainly to Alphaproteobacteria, Betaproteobacteria, Actinobacteria and Bacteroidetes. It is noteworthy that some of the keystone taxa involved in nitrogen cycling are significantly related to total nitrogen, ammonia, temperature and chlorophyll *a*, including *Polaromonas*, *Albidiferax*, SM1A02 and *Leptolyngbya* so on. And the eukaryotic keystone taxa were found in Ascomycota, Choanoflagellida and Heterophryidae. The mutualistic pattern between pro- and eukaryotes was more evident than the competitive pattern. Therefore, it suggests that keystone taxa could be as bio-indicators of aquatic ecosystems.

## 1. Introduction

By adjusting and maintaining the ecological balance in aquatic ecosystems, microorganisms play an indispensable role in the self-purification of waterbodies and could therefore be considered pivotal indicators of the quality of these ecosystems (Hou et al., 2013; Piccini and García-Alonso, 2015; Ma et al., 2018). Changes in the microbial community may have impacts on water quality, such as causing algal blooms and stinking sewers. Environmental factors shape the diversity and composition of microbial communities, which may shift due to spatial–temporal changes in aquatic ecosystems (Mucha et al., 2004; Celussi and Cataletto, 2007; Shu and Gin, 2010; Sun et al., 2014; Jiang et al., 2021). Seasonal temperature changes can be an important factor leading to variations in microbial communities (Liu et al., 2013; Zhu et al., 2019). The spatial–temporal distribution of water properties also has an fundamental influence on microbial communities (Sun et al., 2014; Jiang et al., 2021). Microbial community structure and functions are significantly related to pH in the presence of a surplus of nitrogen in aquatic ecosystems (Joshi et al., 2017). Water eutrophication caused by excess nitrogen and phosphorus lead to an explosion of algae and the consumption of dissolved oxygen (DO; Massey et al., 2020). Chemical oxygen demand (COD_Mn_) and chlorophyll *a* (Chl *a*) are decisive indicators of algal blooms and oxidizable organic pollutants (Massey et al., 2020; Zhang et al., 2020), while Chl *a* and microbial abundance vary seasonally (Li et al., 2017). The significant differences in total nitrogen (TN) and total phosphorus (TP) were found between different regions of a river in a previous study, distinctive effects of TN, DO and Chl *a* on pro- and eukaryotes were also observed in different seasons (Sun et al., 2014). Plankton blooms, including those of cyanobacteria, break out more frequently in warmer spring and summer months than in cooler autumn and winter months (Li et al., 2017; Jiang et al., 2021). Hence, knowledge of the spatial–temporal distribution of microbial communities and water properties will help to predict the quality of aquatic ecosystems (Sun et al., 2014; Jiang et al., 2021).

Some special microbial taxa that dominate nutrient cycles and energy transfers in aquatic ecosystems are highly sensitive to environmental changes, and they are usually may be used as bio-indicators to assess environmental variations in the water quality of rivers and lakes (Azam and Malfatti, 2007; Zeglin, 2015; Guo et al., 2016; Wang et al., 2017; Yang et al., 2019). Microscopic counting has also been applied to determine plankton numbers in the Finnish boreal lakes and Calabar River in Nigeria to screen indicator taxa in those aquatic ecosystems (Lepistö et al., 2004; Uttah et al., 2010). Recently, microbial taxa identified by high-throughput sequencing were used as bio-indicators based on their distinctive responses to disturbance by anthropogenic activities of the Songhua River in China (Yang et al., 2019). However, the identification of bio-indicators in these waterbodies was mainly based on microbial biomass, morphology and abundances. Recent studies have shown that microbial interactions may provide reliable key information on changes for environmental quality, as the reactivity and diversity characteristics of microbial interactions are susceptible to environmental variables (Karimi et al., 2017; Yang et al., 2019). Microbial communities form complex relationships through multiple interactions, so microbial co-occurrence networks can potentially be used to infer coexistence patterns of microbial taxa and thereby to identify keystone taxa in ecosystems (Banerjee et al., 2018; Shi et al., 2020). Microbial keystone taxa have been found to play critical roles in anti-interference in ecosystems and to be involved in traits and functions related to various ecological processes, such as nutrient cycling and carbon degradation (Jiang et al., 2016; Banerjee et al., 2018; Shi et al., 2020). Analyzing the relationship between keystone taxa and environmental factors is a primary method to interpret the interrelations between key biotic and abiotic factors in the ecosystem (Shi et al., 2020; Xu et al., 2021). In terrestrial ecosystems, keystone taxa are significantly influenced by pH and TP, and these environmental factors maintain and regulate functional stability by changing the relative abundances of keystone taxa (Shi et al., 2020; Xu et al., 2021). Similarly, keystone taxa might also be crucial for stability in aquatic ecosystems (Shade and Gilbert, 2015; Xue et al., 2018), and could therefore also be important bio-indicators for water quality. Occasional studies have found relationships between keystone taxa and environmental factors, which are expected to have potential as bio-indicators in aquatic ecosystems. However, the use of keystone taxa as bio-indicators in aquatic ecosystems has not been studied before.

Lake Dongqian, being the typical subtropical freshwater lake, it has been listed as a pilot and model for ecological and environmental lake protection in China due to its great economic and social importance (Jing et al., 2013). Given the ecological importance of microorganisms, taking Lake Dongqian as an example, the temporal–spatial distribution of the community composition of prokaryotes and eukaryotes were analyzed. And define water properties that significantly influence prokaryotic and eukaryotic communities. Besides, the keystone taxa could be used as bio-indicators of water quality were elucidated. It provides a reliable reference for studying the bio-indicators in other lake ecological environment.

## 2. Materials and methods

### 2.1. Study area description and water sample collection

**Figure 1:**
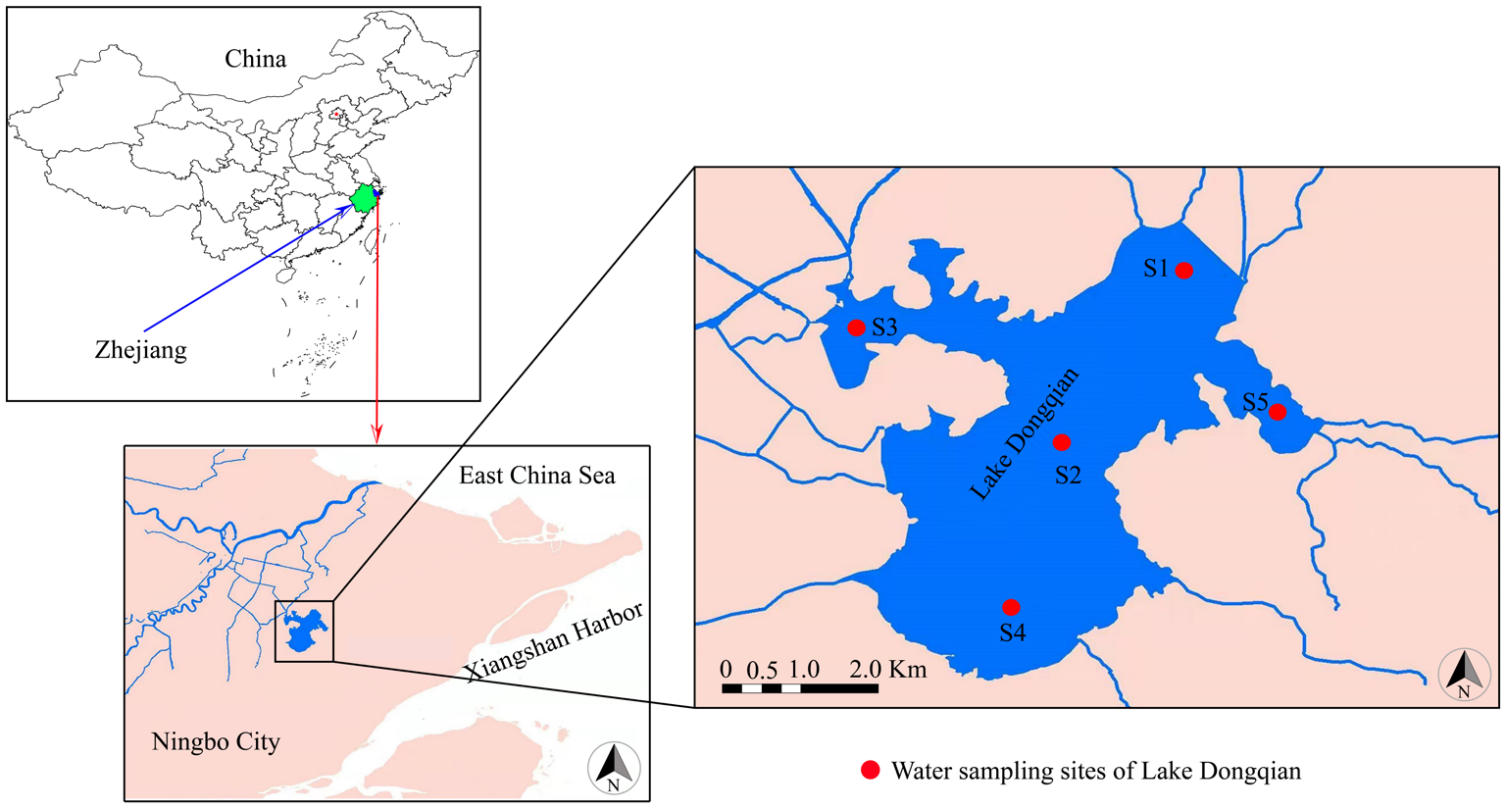
Sampling sites at Lake Dongqian near the city of Ningbo, Zhejiang Province, China.

Lake Dongqian (29°46′ N, 121°39′ E), the largest freshwater lake in Zhejiang Province, is located in the subtropical region of the eastern coastal zone of China (Figure 1). It has a mean depth of 2.2 m, a surface area of 19.91 km^2^, and a water volume of 44.29 million m^3^, and its annual average temperature, frostless period, and precipitation are 15.4 °C, 239 d, and 1,421 mm, respectively (Jing et al., 2013). After conducting long-term fixed-point observations on different monitoring sample plots in Lake Dongqian, five representative water sampling sites were selected. Geographic information on the water sampling sites is presented in Supplementary Table S1; Figure 1. Site 1 was located in the north of the lake, Site 2 was situated in the center of the lake, Site 3 was located in the western area of the lake, Site 4 was positioned in the south of the lake, and Site 5 was situated in the eastern area of the lake. Samples were collected in four seasons, i.e., spring (April), summer (July), autumn (September) and winter (January).

In total, 20 water samples were collected from the five sites in each of the four seasons. For each sample, 1.5 L was collected from a depth of 30 cm under the water surface using a sterilized water sampler (Zhang et al., 2015) and subsequently split into two parts. The first part (0.5 L) was stored in an ice box and quickly transported to the laboratory for DNA analyses, and the second part (1.0 L) was taken to the laboratory within two hours for determination of water properties.

### 2.2. Water property determination

The pH, temperature (Temp), total dissolved solids (TDS), and dissolved oxygen (DO) of the water samples were determined *in situ* by a portable meter (Hach Company, Loveland, CO, United States). Total nitrogen (TN), ammonia (NH_4_^+^), total phosphate (TP), chemical oxygen demand (COD_Mn_) and chlorophyll *a* (Chl *a*) were determined in the laboratory as described previously (Sun et al., 2014; Wang L. et al., 2015).

### 2.3. DNA extraction and qPCR assay

The 0.5 l water samples were filtered through 0.22 μm pore-sized filters (47 mm diameter, Durapore GVWP, Millipore) to collect all microorganisms and then stored at −80 °C for DNA extraction. DNA extraction was performed as described previously (Zhang et al., 2015) using the Fast DNA® Spin Kit for Soil (MP Biomedicals, LLC, Illkirch, France). The total DNA obtained was collected in 50 μL of DES solution (Qbiogene, Vista, CA, United States), and its concentration and purity were determined with a Nanodrop spectrophotometer.

qPCRs were performed for the 16S rRNA and 18S rRNA genes according to the method described previously (Zhang and Laanbroek, 2020). All samples were analyzed in triplicate. The qPCR conditions are listed in Supplementary Table S2. The primer set for amplification of the 16S rRNA gene was 338F/806R (Zhang and Laanbroek, 2020), and the primer set for amplification of the 18S rRNA gene was 817F/1196R (Zhang and Laanbroek, 2020). The obtained amplification efficiency was between 95 and 103%, the R^2^ value was between 0.99–1.00, and the slope ranged from −3.0 to −3.4. A negative control with no template DNA was included in the qPCR assay. Product specificity was confirmed by melting curve analysis and visualization in 1.0% agarose gels. No significant inhibitors were found in the DNA extract.

### 2.4. High-throughput sequencing and sequence analysis

For the amplification and sequencing methods of the 16S rRNA and 18S rRNA genes, we referred to previously published studies on water microbes (Jiang et al., 2021). All samples were analyzed in triplicate. The primer sets for the 16S rRNA and 18S rRNA genes were 338F/806R (Zhang and Laanbroek, 2020) and 817F/1196R (Zhang and Laanbroek, 2020), respectively, both with attached barcode sequences (Gupta et al., 2019). The PCR conditions are listed in Supplementary Table S2. Sequencing was carried out by Shanghai Majorbio Bio-pharm Technology (Shanghai, China) using an Illumina MiSeq PE300 platform (Illumina, United States).

Sequence analysis was performed with the free online Majorbio I-Sanger cloud platform.^1^ For the 20 water samples, Trimmomatic was used to identify and remove chimeric sequences, and FLASH was then used for sequence splicing (Zhang and Laanbroek, 2020). Then, the optimized sequences were clustered into operational taxonomic units (OTUs) with similarities of 97% or greater using UPARSE 7.1 (Liu et al., 2015). A valid sequence alignment was performed in the SILVA (Release138 http://www.arb-silva.de) database, and OTUs were subsequently identified to the phylum, class, order, family and genus levels using the Ribosomal Database Project with the Bayesian classifier at a 70% threshold (Jiang et al., 2021). The Mothur software package was used to determine the alpha diversity indices of the microbial communities, including the Ace, Chao1, Shannon, Simpson and coverage indices. The overlap in OTUs in the different water samples was analyzed using the VennDiagram package (Xue et al., 2018). Principal coordinates analysis (PCoA) created by QIIME 1.7.0 was combined with permutational multivariate analyses of variance (PERMANOVA) to test for microbial dissimilarities between the different seasons (Zhang and Laanbroek, 2020). The relationship between microbial community structures and water properties was shown by redundancy analysis (RDA) offered by the Canoco 4.5 software package (Yang et al., 2019). To detect significantly different microbial taxa between the four seasons, a linear discriminant analysis (LDA) effect size (LEfSe) threshold score of 4.0 and a significant *α* of 0.05 were applied to detect distinctive microbial taxa with a comparison of all-against-all styles (Zhang and Laanbroek, 2020).

### 2.5. Microbial network analysis

Four seasonal co-occurrence networks of pro- and eukaryotic genera based on Pearson correlations were constructed online using the Molecular Ecological Network Analyses Pipeline (MENAP; Deng et al., 2012). To demonstrate that the constructed co-occurrence networks were non-random, random networks that were constructed using appropriate thresholds calculated by random matrix theory, served as controls (Deng et al., 2012). The within-module connectivity (*Zi*) and among-module connectivity (*Pi*) and other network topological properties in co-occurring networks used to identify peripherals, connectors, module hubs and network hubs were derived from MENAP online analysis (Deng et al., 2012). The correlation network was analyzed by the vegan and igraph packages in R version 4.2.0,^2^ while a Spearman’s correlation coefficient (ρ) larger than 0.6 was assumed to be statistically significant (*p* value <0.05; Barberán et al., 2012).

### 2.6. Statistical analysis

Principal component analysis (PCA) was used to analyze the changes in water properties at different sites in the four seasons with the PAST ver.2.17c software package (Zhang et al., 2015). Spearman rank-order analysis (2-tailed) of IBM SPSS Statistics 24 software was used to analyze the correlation between water properties. Chord diagrams of the associations of microbial taxa with different seasons were drawn using the ggplot2 and circlize packages of R version 4.2.0 (see text footnote 2) (Zhao et al., 2022). Gephi software was used to generate the layout and visualization of all networks (Zhou et al., 2020).

## 3. Results

### 3.1. Water properties

**Figure 2:**
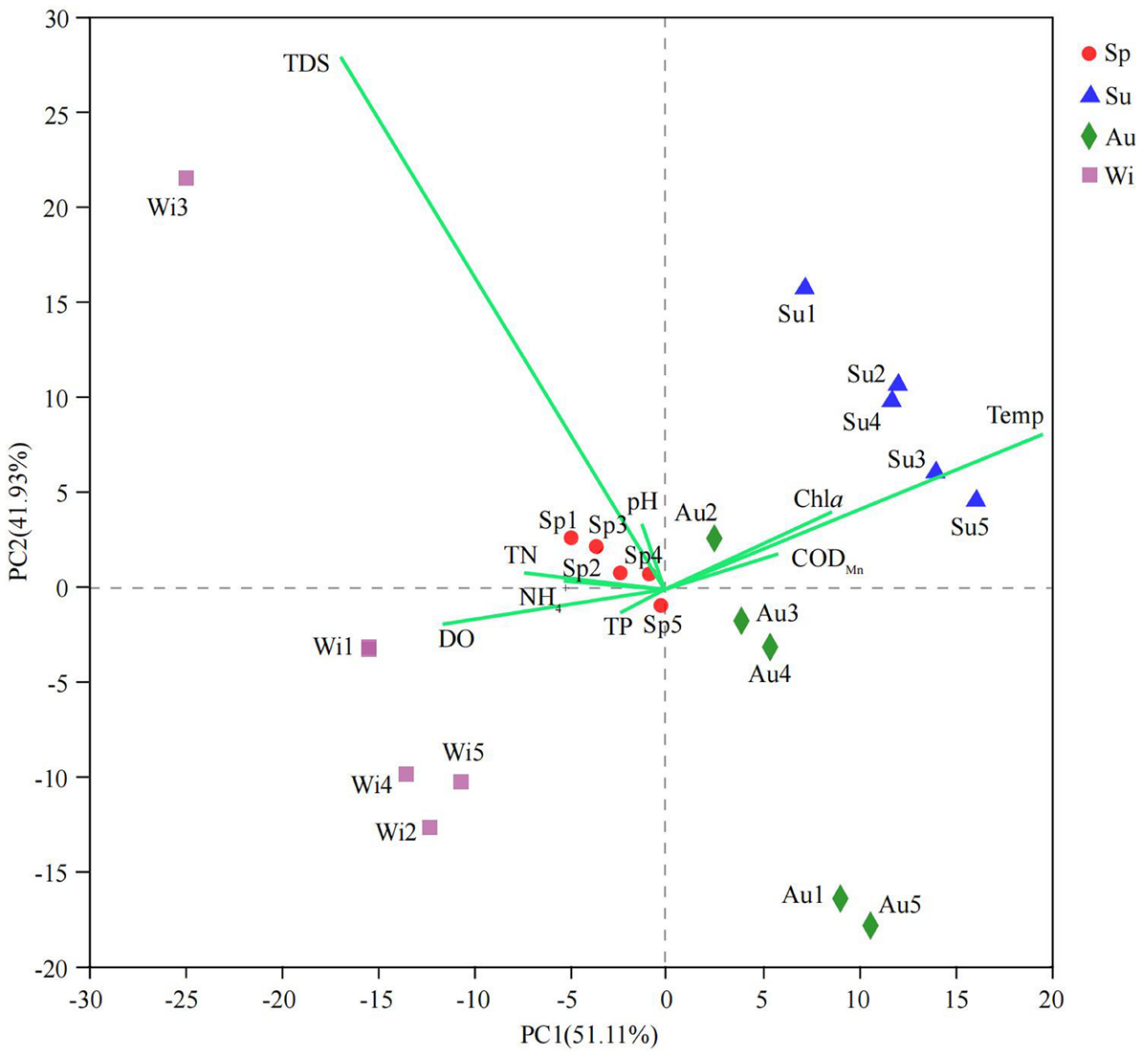
Biplot of the results of a principal-component analysis (PCA) on water properties obtained in four seasons at five different water sampling sites in Lake Dongqian. Sp, Su, Au, and Wi represent spring, summer, autumn and winter, respectively, whereas Sp1-Sp5, Su1-Su5, Au1-Au5, and Wi1-Wi5 represent samples collected at the different sampling sites in spring, summer, autumn and winter, respectively.

Water properties in Lake Dongqian varied with seasons and locations (Supplementary Table S3). PCA was performed on water properties, and the first two principal components explained 93.04% of the total variance between the samples (Figure 2). The sites in spring and winter showed higher values for pH, TN, NH_4_^+^, TP, DO, and TDS, while the sites in summer and autumn displayed higher values for Temp, COD_Mn_, and Chl *a*. The water properties in spring were more similar between the sites than in the other seasons, while the differences in the water properties between the sites in autumn and winter were larger than those in spring and summer. The results of Spearman rank-order correlation analyses showed several significant correlations. Temp was positively correlated with Chl *a* and negatively correlated with TN, NH_4_^+^ and DO. pH was positively correlated with TDS and negatively correlated with DO. TN was positively correlated with NH_4_^+^ and negatively correlated with COD_Mn_. TP and DO were negatively correlated with COD_Mn_ and Chl *a,* respectively (Supplementary Figure S1).

### 3.2. Abundance and *α*-diversity of microorganisms

The abundances of the 16S and 18S rRNA genes varied with season and site (Table 1). For the prokaryotes, clear differences in abundance were observed between different sites in spring, while these differences gradually decreased from spring to summer, autumn and winter. Contrary to the prokaryotes, the difference in the abundance of eukaryotes between different sites became gradually larger over time from spring to winte. The abundance of the 18S rRNA gene was higher than that of the 16S rRNA gene in all seasons except summer, especially in autumn, the abundance of 16S rRNA gene was significantly higher than 18S rRNA gene (Supplementary Table S4).

**Table 1:** Genes abundance in Lake Dongqian in spring, summer, autumn, and winter.

Sampling season	Sampling site	Gene abundance (copy number·L^−1^ water)	
		16S rRNA gene	18S rRNA gene
Spring	Sp1	1.85 × 10^7^	1.13 × 10^8^
	Sp2	1.28 × 10^8^	6.18 × 10^7^
	Sp3	9.47 × 10^6^	7.17 × 10^8^
	Sp4	1.09 × 10^7^	1.03 × 10^8^
	Sp5	1.18 × 10^7^	7.03 × 10^7^
Summer	Su1	1.06 × 10^7^	6.72 × 10^6^
	Su2	7.91 × 10^5^	1.07 × 10^7^
	Su3	8.29 × 10^6^	8.31 × 10^6^
	Su4	5.83 × 10^6^	1.21 × 10^6^
	Su5	6.22 × 10^6^	1.27 × 10^6^
Autumn	Au1	1.75 × 10^7^	1.44 × 10^10^
	Au2	7.70 × 10^6^	9.93 × 10^8^
	Au3	1.38 × 10^7^	8.96 × 10^9^
	Au4	1.04 × 10^7^	1.91 × 10^8^
	Au5	5.16 × 10^6^	3.83 × 10^8^
Winter	Wi1	3.22 × 10^6^	4.04 × 10^9^
	Wi2	7.49 × 10^6^	9.51 × 10^8^
	Wi3	8.12 × 10^6^	2.34 × 10^6^
	Wi4	8.29 × 10^6^	2.44 × 10^10^
	Wi5	9.94 × 10^6^	7.77 × 10^6^

Sampling sites: Sp1-Sp5, Su1-Su5, Au1-Au5, and Wi1-Wi5 represent five different sampling sites in spring, summer, autumn and winter.

**Figure 3:**
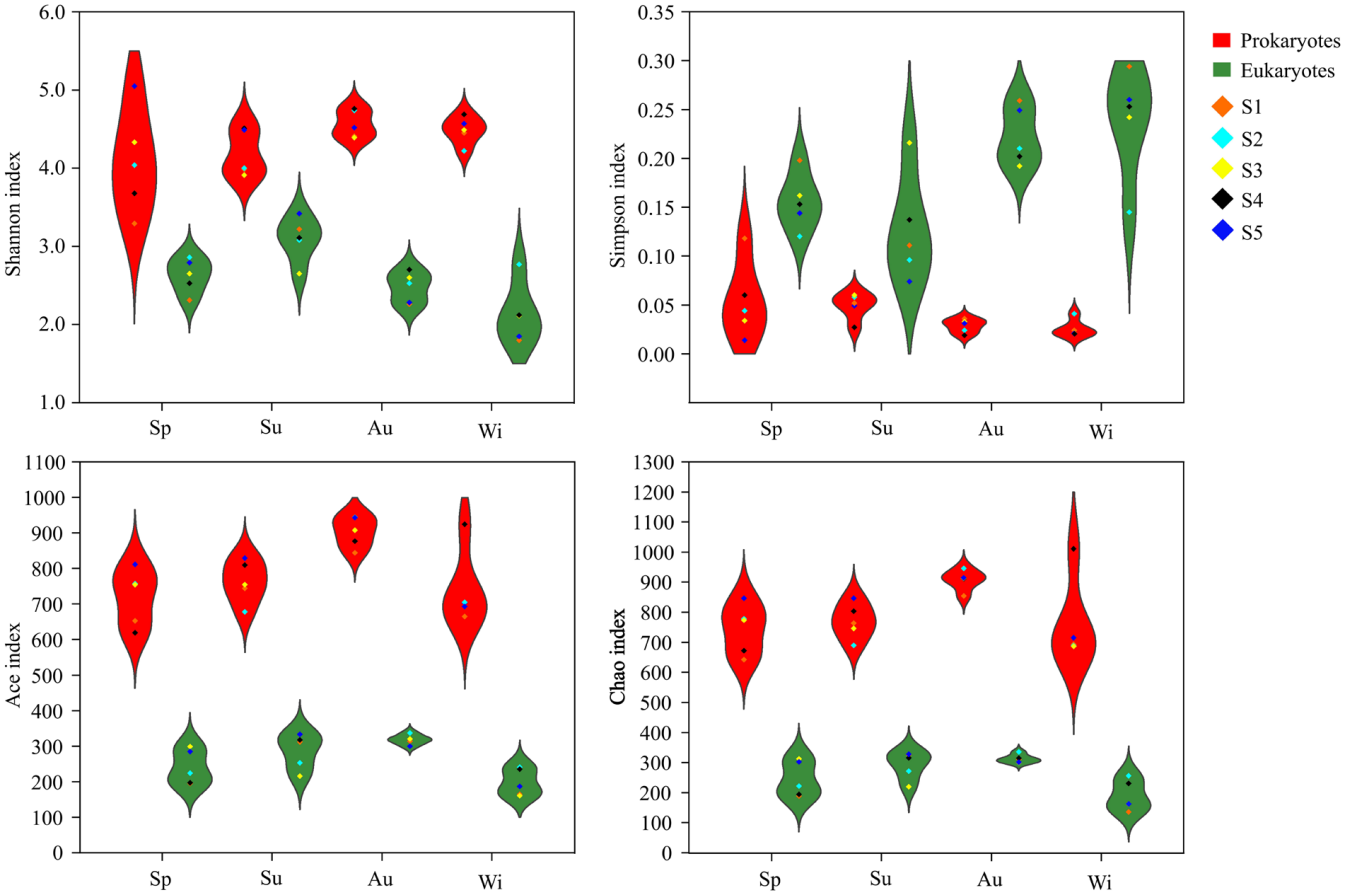
Violin plot of the microbial α-diversity indices in Lake Dongqian at four seasons. Sp, Su, Au and Wi represent spring, summer, autumn and winter, respectively. S1, S2, S3, S4 and S5 represent the five sampling sites.

The coverage of the 16S and 18S rRNA genes was greater than 99%, indicating that the sequencing depth covered most of the microorganisms. The Shannon, Ace and Chao1, but not Simpson, indices of prokaryotes in the different seasons were significantly higher than those of eukaryotes, indicating that the diversity and richness of the prokaryotes were higher than those of the eukaryotes (Figure 3; Supplementary Table S4).

### 3.3. Community compositions of pro- and eukaryotes

**Figure 4:**
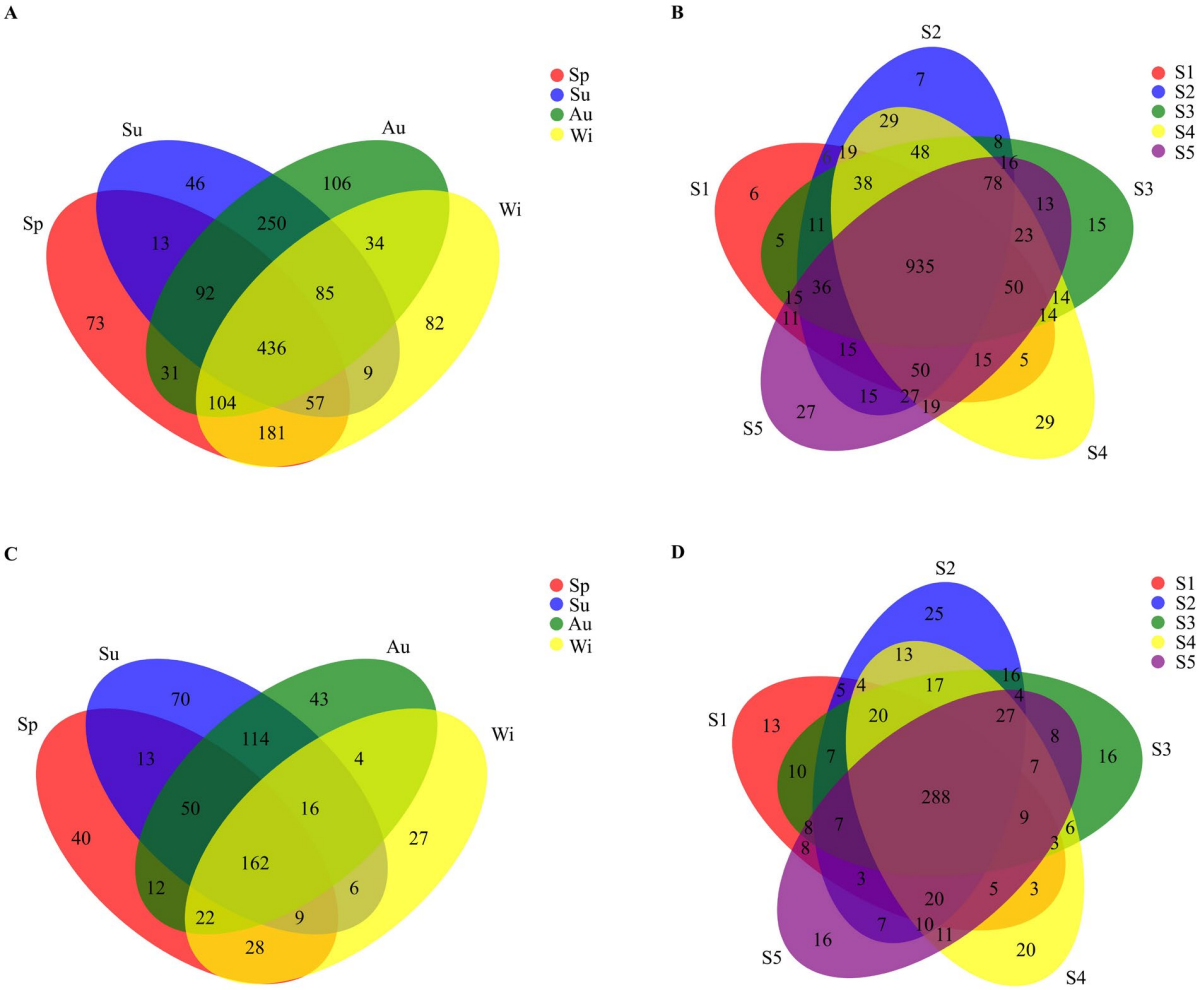
Venn diagrams of the OTU distribution of prokaryotes **(A,B)** and eukaryotes **(C,D)** for seasons **(A,C)** and sampling sites **(B,D)**. Sp, Su, Au, and Wi represent spring, summer, autumn and winter, respectively. S1, S2, S3, S4, and S5 represent the five sampling sites.

A total of 1,599 different OTUs of the 16S rRNA gene were found across all samples. The number of shared OTUs in all seasons was 436 (27.3%; Figure 4A), while the number of shared OTUs at all sampling locations reached 935 (58.5%; Figure 4B). A total of 616 different OTUs of the 18S rRNA gene were found in all samples; 162 (26.3%) were shared by all seasons (Figure 4C), while the number of shared OTUs at all sampling locations reached 288 (46.8%; Figure 4D). Therefore, the contribution of seasons to the distribution of pro- and eukaryotic taxa was higher than that of sampling locations. For the prokaryotes, the phylum Actinobacteria was dominant in the summer and autumn samples, while the dominant phyla in spring and winter were more variable at the different sites (Supplementary Figure S2). In spring, Proteobacteria (in samples Sp1 and Sp2), Cyanobacteria (in samples Sp3 and Sp4) and Actinobacteria (in sample Sp5) were the dominant phyla. In winter, the phylum Actinobacteria was dominant in samples Wi1 and Wi2, while the phylum Proteobacteria was most prominent in samples Wi3, Wi4 and Wi5. Betaproteobacteria was significantly more abundant in winter than in the other seasons. For the eukaryotes, the phyla Ciliophora (in samples Sp1, Sp4 and Sp5), P1-31 (in sample Sp3) and Kathablepharidae (in sample Sp2) were dominant at the different sites in spring (Supplementary Figure S2). In summer, Chytridiomycota was the dominant phylum in samples Su1, Su2 and Su3, while P1-31 and Choanoflagellida were more prominent in samples Su4 and Su5, respectively. The phylum Cryptomonadales was dominant in autumn and in samples Wi1, Wi2 and Wi3 in winter. The relative abundance of Ciliophora in samples Wi4 and Wi5 surpassed that of Cryptomonadales in winter.

**Figure 5:**
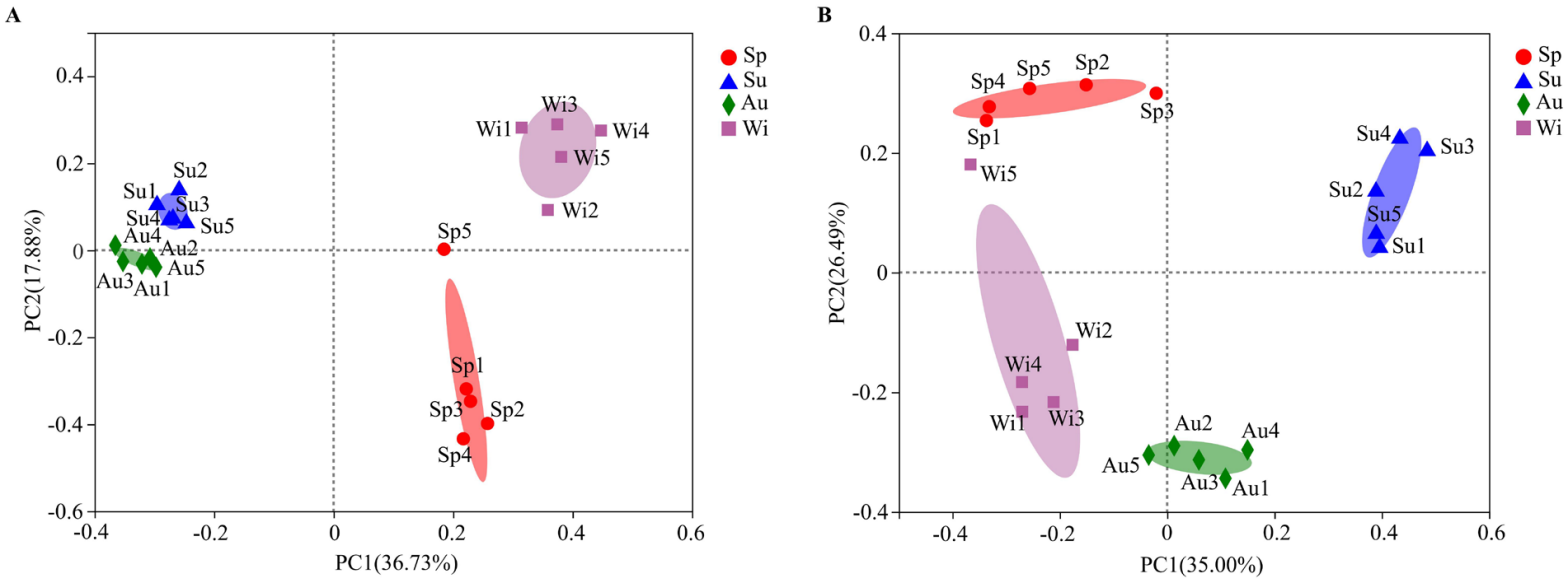
Biplot of the results of a principal co-ordinates analysis (PCoA) of OTUs based on the 16S rRNA gene **(A)** and 18S rRNA gene **(B)** detected in four seasons at different sampling sites at Lake Dongqian. Sp, Su, Au, and Wi represent spring, summer, autumn and winter, respectively, and Sp1-Sp5, Su1-Su5, Au1-Au5, and Wi1-Wi5 represent samples collected at the different sampling sites in spring, summer, autumn and winter, respectively.

PERMANOVA showed that both prokaryotic (*R*^2^ = 0.8737, *p* = 0.001) and eukaryotic (*R*^2^ = 0.8273, *p* = 0.001) community compositions were significantly affected by season. For the prokaryotes, the first two axes of the PCoA explained 54.61% of the variance (Figure 5A). The composition of the prokaryotic communities at the different sampling sites was most similar in summer and autumn. Compared to summer and autumn, the prokaryotic community varied greatly between the sites in spring and winter. For the eukaryotes, the first two axes of a PCoA together explained 61.49% of the total variance (Figure 5B). The composition of the eukaryotic community was also most similar in summer and autumn. The composition of the eukaryotic community in spring and winter was most different. Overall, the seasonal variations in pro- and eukaryotic communities were larger than the spatial variations.

Distinctive pro- and eukaryotic taxa appeared in the different seasons, as shown in the cladograms (Supplementary Figures S4A,B, S5A,B). LDA scores of 4.0 or greater were confirmed by LEfSe (Supplementary Tables S5A,B, S6A,B). The relative numbers of distinctive prokaryotic taxa were largest in winter, accounting for 42.31% of the total number of distinctive prokaryotic taxa, whereas the relative numbers were lowest in summer, accounting for only 11.54% of the total number of distinctive prokaryotic taxa. The relative numbers of distinctive eukaryotic taxa were largest in spring and summer, accounting for 30.77% of the total distinctive eukaryotic taxa. With 15.38% of the total distinctive eukaryotic taxa, winter had the lowest relative number of distinctive eukaryotic taxa.

### 3.4. Network analysis of microbial communities and keystone taxa

**Figure 6:**
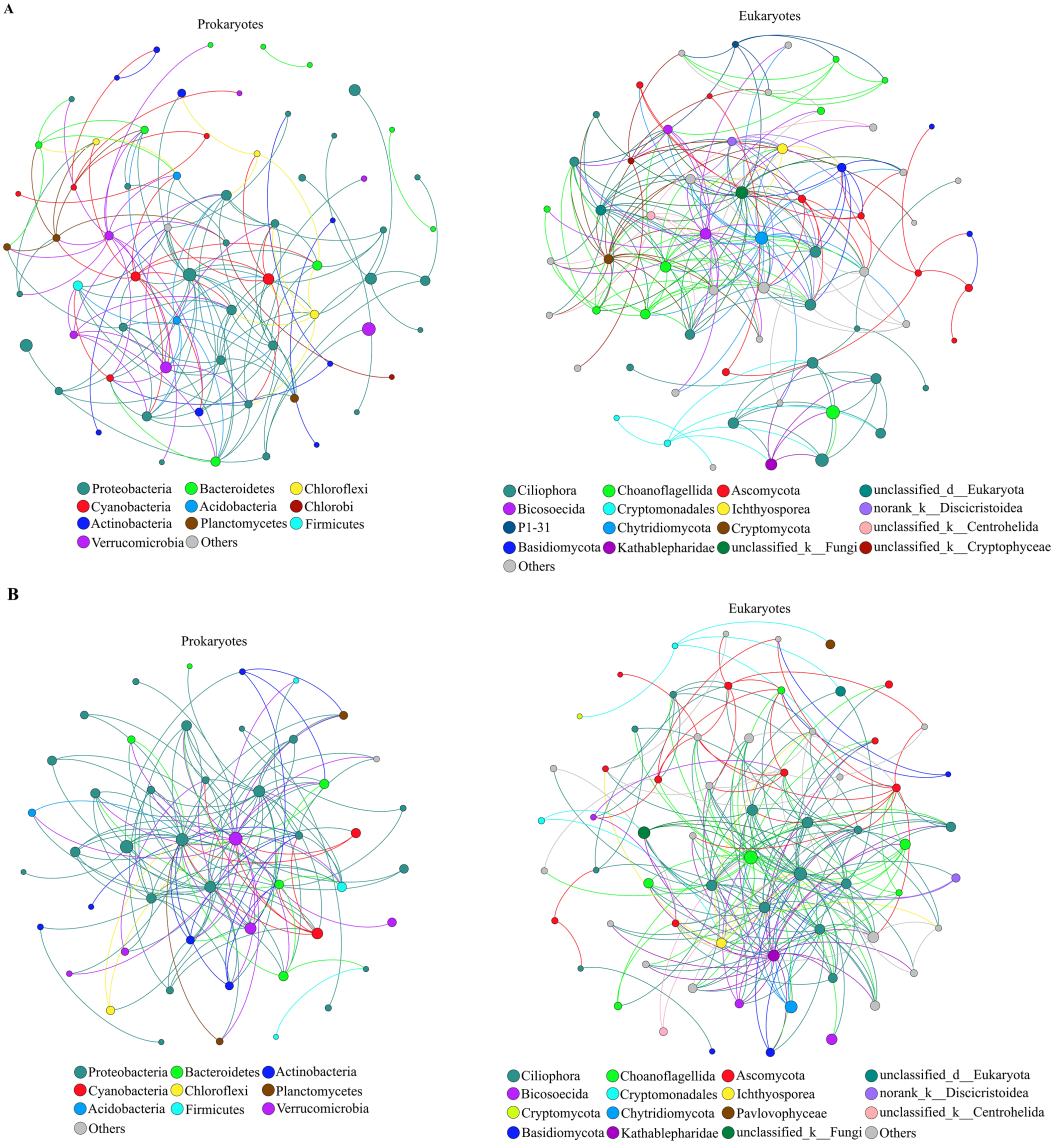
Positive **(A)** and negative **(B)** co-occurrence networks of prokaryotic and eukaryotic communities (at the genus level) in Lake Dongqian throughout the year. Each node signifies a genus, colors of the nodes indicate different phyla; phyla with relative abundance <1% are merged and presented as others and shown in gray. The size of each node is proportional to the number of edges (i.e., degree).

Based on all high-throughput data collected throughout the year, a co-occurrence network of pro- and eukaryotic communities was constructed at the genus level (Figures 6A,B). The obtained values for the harmonic geodesic distance (HD), the average clustering coefficient (avgCC) and the modularity of both pro- and eukaryotic networks were higher than those obtained with corresponding random networks constructed with the same number of nodes, which indicates that the networks were non-random (Table 2). The average connectivity (avgK), avgCC and modularity of prokaryotic networks were smaller than those of eukaryotic networks, while HD was longer for prokaryotes, indicating that prokaryotic networks were less complex and stable than eukaryotic networks. In the prokaryotic networks, the positive interactions between nodes were stronger than the negative interactions, whereas the opposite was true in the eukaryotic networks (Table 2; Figures 6A,B). Correlation networks were used to analyze the coexistence patterns of pro- and eukaryotes in the different seasons (Supplementary Figure S6). In spring, there were 603 positive and 65 negative links in the pro- and eukaryotic correlation networks, 559 positive and 101 negative links in summer, 364 positive and 88 negative links in autumn, and 712 positive and 108 negative links in winter. Hence, based on the total number of links, the relationship between pro- and eukaryotic taxa was strongest in winter and weakest in autumn. In addition, based on the ratio between positive and negative links, the mutualistic relationship between pro- and eukaryotic taxa was more obvious than the competitive relationship in all seasons.

**Table 2:** Comparison between topological properties of empirically obtained molecular ecological networks (MENs) between prokaryotes and eukaryotes communities (at the genus level) throughout the year and the associated random MENs in Lake Dongqian.

Microorganisms	Empirical networks									Random networks		
	Similarity threshold (St)	Total nodes	Total links	Positive links	Negative links	Average connectivity (avgK)	Harmonic geodesic distance (HD)	Average clustering coefficient (avgCC)	Modularity (M)	Harmonic geodesic distance (HD)	Average clustering coeffificient (avgCC)	Modularity (M)
Prokaryotes	0.87	77	298	158	140	7.740	2.267	0.179	0.268	2.145 ± 0.032	0.167 ± 0.020	0.232 ± 0.009
Eukaryotes	0.57	78	454	224	230	11.641	2.061	0.370	0.278	2.148 ± 0.025	0.326 ± 0.013	0.175 ± 0.008

**Figure 7:**
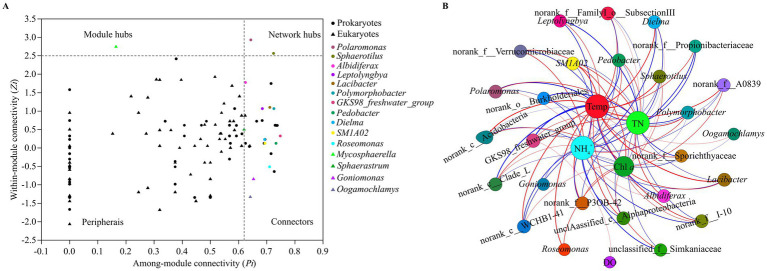
Zi-Pi plot **(A)** showing the distribution of prokaryotic and eukaryotic genera based on their module-based topological roles. Dots represent prokaryotic genera and triangles represent eukaryotic genera. The topological role of each genus is determined according to the scatter plot of within-module connectivity (*Zi*) and among-module connectivity (*Pi*), the threshold values of *Zi* and *Pi* for categorizing genera are 2.5 and 0.62, respectively. Identified genera are indicated by different colors. Correlation network graph **(B)** for significant relationships between water properties and microbial keystone taxa in Lake Dongqian. The size of each node is proportional to the number of edges (i.e., degree). A red edge indicates a significant positive correlation (*p* < 0.05) between two individual nodes, while a blue edge indicates a significant negative correlation (*p* < 0.05).

The prokaryotic taxa that were identified in the network hubs, module hubs and connectors of the prokaryotic networks included the genera GKS98 *freshwater group*, *Pedobacter*, *Polymorphobacter*, *Sphaerotilus*, *Lacibacter*, *Roseomonas*, SM1A02, *Dielma*, *Leptolyngbya*, *Polaromonas* and *Albidiferax*, which mainly belong to Alphaproteobacteria, Betaproteobacteria, Actinobacteria and Bacteroidetes (Figure 7A). The eukaryotic taxa that were identified in the network hubs and connectors of the eukaryotic networks included the genera *Mycosphaerella*, *Goniomonas*, *Oogamochlamys*, and *Sphaerastrum,* which belong to the phyla of Ascomycota, Choanoflagellida and Heterophryidae. Network hubs, module hubs and connectors are considered keystone taxa in microbial networks. The correlation network showed that many keystone taxa were significantly affected by TN, NH_4_^+^, Temp, DO and Chl *a* (Figure 7B). The degree values of NH_4_^+^ and Temp in the network were both 25, and the degree values of TN, Chl *a* and DO were 24, 18 and 1, respectively. Hence, NH_4_^+^ and Temp significantly affected most keystone taxa, followed by TN and Chl *a*, while only one keystone taxa was significantly affected by DO. Moreover, the distribution of keystone taxa in pro- and eukaryotic networks differed significantly across the four seasons (Supplementary Figures S7A,B).

### 3.5. Relationship between microbial community compositions and water properties

**Figure 8:**
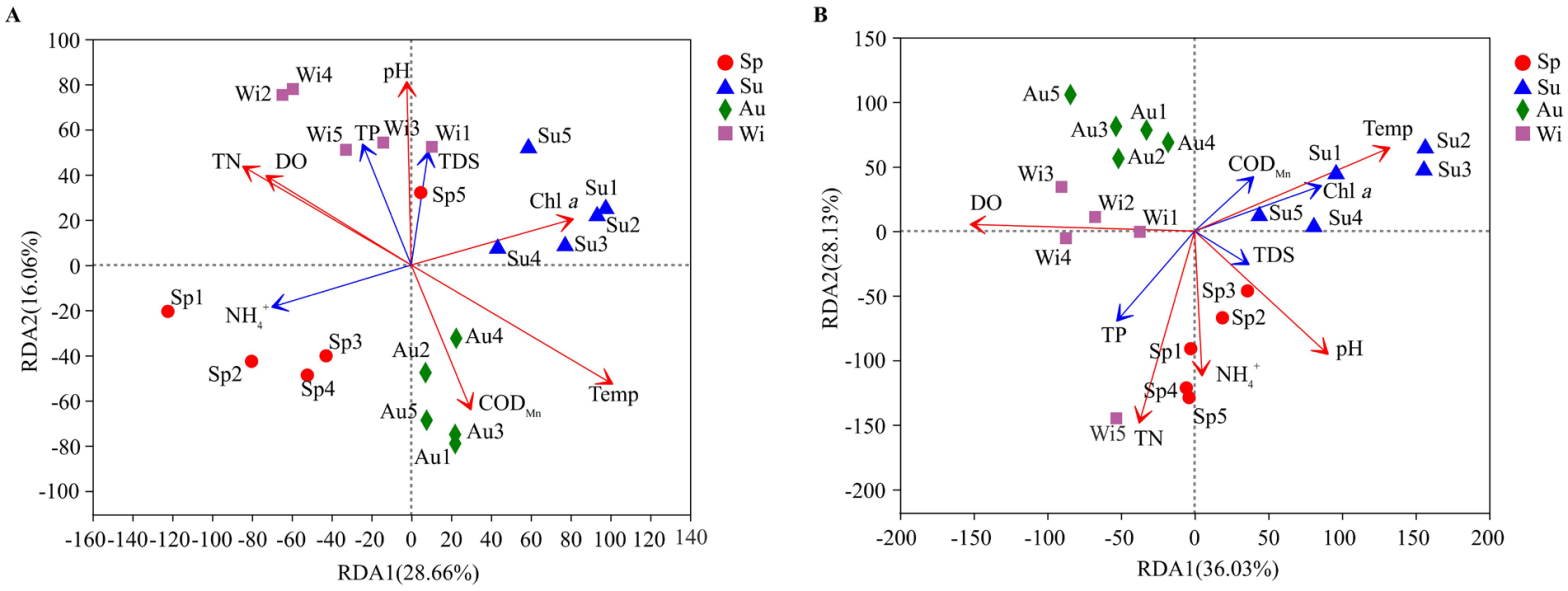
Biplot of a redundancy analyses (RDA) between water properties and prokaryotic **(A)** and eukaryotic **(B)** communities (at the genus level) observed in samples collected from Lake Dongqian. Sp, Su, Au, and Wi represent spring, summer, autumn and winter, respectively; Sp1-Sp5, Su1-Su5, Au1-Au5, and Wi1-Wi5 represent samples collected at the different sampling sites in spring, summer, autumn and winter. The *R* value is displayed in different colors of the arrows (red arrow: *p* < 0.05, blue arrow: *p* ≥ 0.05).

At the genus level, redundancy analysis (RDA) was used to analyze the impact of water properties on the microbial community composition. Of the cumulative variance observed in the RDA on the prokaryotes, 44.72% was explained by the first two axes of the RDA biplot (Figure 8A). Temp, pH, TN, DO, COD_Mn_ and Chl *a* had a significant effect on the prokaryotic community composition (0.001 ≤ *p* < 0.05). Of the cumulative variance observed in the RDA on the eukaryotes, 65.49% was explained by the first two axes of the RDA biplot (Figure 8B). Temp, pH, TN, NH_4_^+^ and DO had a significant impact on the eukaryotic community composition (0.001 ≤ *p* < 0.05). Except for COD_Mn_, Chl *a* and TDS, water properties had a greater impact on the eukaryotic community than on the prokaryotic community.

## 4. Discussion

Since the pro- and eukaryotes tended to cluster preferentially in the different seasons, their communities seem to respond to seasons than to sites. More prokaryotic than eukaryotic keystone taxa, while the interactive links between prokaryotic taxa were simpler than those between eukaryotes. Interestingly, quite a few of the microbial keystone taxa are potentially involved in the nitrogen cycle, and they are also closely related to TN and NH_4_^+^, which are critical parameters of water quality. Although there have been a number of studies on bio-indicators in lakes and rivers, they usually used microbial biomass, morphology and numbers as the basis for bio-indicators (Lepistö et al., 2004; Uttah et al., 2010). None of these previous results included microbial keystone taxa that are responsible for maintaining the stability and function of ecosystems. In contrast, our study confirmed the correlations between microbial keystone taxa at the genus level and major environmental factors, and suggests that microbial keystone taxa could be used as bio-indicators of water quality in lakes such as Lake Dongqian.

### 4.1. Contrasting interactions between pro- and eukaryotic communities

Co-occurrence networks reflect the coexistence pattern of microbial taxa. Positive correlations imply mutually beneficial interactions and niche overlap between taxa, while negative correlations represent mutual competition for resources (Deng et al., 2012; Faust and Raes, 2012; Zheng et al., 2017). Compared with the prokaryotic network in Lake Dongqian over the whole year, the corresponding eukaryotic network had higher avgK and avgCC and shorter HD values (Table 2), indicating that interactions between eukaryotic taxa were stronger than those between prokaryotic taxa. This has been shown previously for microbial networks in soils (Hou et al., 2019) but is new to our knowledge of aquatic ecosystems. The positive correlations between prokaryotic taxa were stronger than the corresponding negative correlations; conversely, the positive correlations between eukaryotic taxa were slightly weaker than the negative correlations (Figures 6A,B), suggesting that prokaryotic interactions were more mutualistic than antagonistic, thereby distinguishing them from eukaryotic taxa in Lake Dongqian. The closest interactions between pro- and eukaryotes were found in winter, while the weakest occurred in autumn. The positive interactions always dominated the negative interactions between pro- and eukaryotes in the aquatic ecosystem during the whole year. Their coexistence, which is regulated by environmental conditions, remains generally stable through mutually beneficial interactions. Competition for resources would intensify and maintain network coseismic displacement and stability once substance resources for survival were under high nutrient conditions, thereby maintaining the ecosystem balance (Mitri et al., 2016; Zhou et al., 2020).

### 4.2. Microbial keystone taxa and their relationship to environmental variables

Network hubs, module hubs and connectors in microbial networks represent keystone taxa that maintain the stability and functions of microbial communities (Karimi et al., 2017; Shi et al., 2020). There were more pro- than eukaryotic keystone taxa in the microbial networks of Lake Dongqian (Figure 7A), which suggests that more important prokaryotic taxa might be closer to aquatic ecosystem than those of the eukaryotes. Both NH_4_^+^ and Temp were considered the most primary factors in the correlation network, followed by TN and Chl *a*, and all had significant effects on the keystone taxa (Figure 7B). It has been suggested that these factors are the most central for the regulation of microbial community composition patterns and for the spatial–temporal distribution of microbial taxa in aquatic ecosystems (Liu et al., 2013; Zhu et al., 2019). The ratio between pro- and eukaryotes fluctuated with the variation in NH_4_^+^ and TN values, which are indicative of the water quality and are likely to be decisive for maintaining the balance in aquatic ecosystems. An excess of NH_4_^+^ and TN could readily lead to blooms of planktonic algae, resulting in a sharp decrease in the ratio between pro- and eukaryotes once these nutrients become beyond the threshold value of water safety (Chen et al., 2018; Massey et al., 2020). Chl *a* is also closely related to algal blooms in water, and its drastic changes in concentration imply that the stability of the aquatic ecosystem has been damaged (Massey et al., 2020; Zhang et al., 2020). The co-occurrence patterns of microbial taxa in aquatic ecosystems have revealed that environmental factors regulate the functional stability of keystone taxa by interfering with and changing their abundance (Shade and Gilbert, 2015; Xue et al., 2018). By interacting with TN, NH_4_^+^, Temp, and Chl *a,* the keystone taxa will determine the structure and functions of microbial networks and therefore of the whole ecosystem of Lake Dongqian.

Hence, microbial keystone taxa probably regulate the water quality in Lake Dongqian. For example, *Sphaerotilus* which can effectively purify water from organic pollutants (Solisio et al., 2000), was significantly positively affected by Temp and Chl *a*, but inhibited by TN and NH_4_^+^. *Polaromonas* and *Albidiferax*, which are denitrifiers enriched from eutrophic waters and from water with a high nitrate content (Xu et al., 2019; Tian and Wang, 2021), respectively, showed significant negative correlations with both TN and NH_4_^+^ in Lake Dongqian. SM1A02, which is a type of anammox bacterium (Ding et al., 2018), also showed significant negative correlations with both TN and NH_4_^+^ in Lake Dongqian. *Leptolyngbya*, which is a well-known genus of Cyanobacteria associated with nitrogen fixation, primary productivity, and algal blooms (Li et al., 2020), was significantly and positively correlated with Temp, TN and NH_4_^+^ in Lake Dongqian. Therefore, we suggest that most of the keystone taxa can be used as bio-indicators to assess the water quality together with the abiotic indicators in Lake Dongqian since they were significantly related to a number of water variables and played key ecological functions.

### 4.3. Contrasting temporal trends between pro- and eukaryotic community compositions

The number of unique pro- and eukaryotic taxa specific to each of the seasons was larger than that found for each of the sampling sites in Lake Dongqian (Figure 4). This indicates that the specificity of pro- and eukaryotic taxa based on seasonal variation was greater than that based on spatial differences. Actinobacteria and Proteobacteria dominated in all seasons, while the dominance of eukaryotic taxa such as Cryptomonadales, Ciliophora, Chytridiomycota, P1-31, Kathablepharidae and Choanoflagellida was more variable over time. This is consistent with studies performed in the Danjiangkou Reservoir in China, indicating that eukaryotic communities are more variable in time than prokaryotic communities (Zhang et al., 2021). Compared with the other three seasons, Betaproteobacteria preferred to grow in winter in Lake Dongqian, which was similar to previous studies in Lake Redon in the Pyrenees in Spain and in Lake Témpanos of Queulat National Park in Chile (Llorens-Marès et al., 2011). Apparently, the conditions in winter are more favorable for the survival and reproduction of Betaproteobacteria than of other classes of Proteobacteria (Llorens-Marès et al., 2011; Aguayo et al., 2017). Furthermore, the similarities in pro- and eukaryotic community compositions at the different sites within the same season were higher than those for one site between the seasons (Figures 5A,B). The influence of seasons on the microbial community composition was greater than that of locations, which is consistent with previous studies in the Qingcaosha Reservoir, the Pearl River and the Jiulong River, all in China (Wang Y. et al., 2015; Li et al., 2017; Jiang et al., 2021). Temperature is the main driving force for the seasonal succession of the microbial community in waters (Liu et al., 2013; Zhu et al., 2019), as it promotes microbial growth in summer and autumn more than in spring and winter. TN was more favorable for pro- and eukaryotic community compositions in spring and winter than in summer and autumn, while NH_4_^+^ had a stronger positive influence on the eukaryotic community composition in spring, which is similar to earlier studies (Liu et al., 2013; Sun et al., 2014; Zhu et al., 2019). High DO with low temperature in winter was more favorable to both prokaryotic and eukaryotic communities (Sun et al., 2014). Chl *a* only had a positive effect on the prokaryotic community composition in summer, which might be due to the close correlation between Chl *a* and cyanobacteria in this season (Sun et al., 2014).

### 4.4. Contrasting microbial taxa with statistically significant differences among seasons

The highest and lowest numbers of distinctive prokaryotic taxa occurred in winter and summer, respectively, while eukaryotic taxa were more homogeneous than prokaryotes throughout the year (Supplementary Tables S5A,B, S6A,B). This indicates that seasonal succession had more effect on the prokaryotes than on the eukaryotes in Lake Dongqian. Since the distinctive pro- and eukaryotic taxa showed a mutual inhibitory relationship in Lake Dongqian at the moments when prokaryotic taxa dominated, the prokaryotic taxa inevitably influenced the survival of the eukaryotic taxa (Bucci et al., 2014; Zhou et al., 2020). Distinctive pro- and eukaryotic taxa were enriched in the different seasons and played pivotal roles in Lake Dongqian. For instance, it has been shown that *Microcystis,* which accumulates in spring, is critical for the microbial community structure in aquatic ecosystems (Zuo et al., 2021). The eukaryotic taxon *Tintinnidium,* which feeds on most bacteria, is an important biotic factor in regulating the bacterial community in aquatic ecosystems (Hisatugo et al., 2014). The prokaryotic taxon HgcI clade that was found in summer could improve water quality due to its participation in N fixation and denitrification (Ruprecht et al., 2021). The CL500-29 marine group, which was found in autumn, can degrade dissolved organic carbon (Lindh et al., 2015). Bacilli are cardinal microorganisms for the degradation of pollutants and for ecological restoration (Teles et al., 2018). *Flavobacterium*, *Rhodoferax*, *Rhodobacter*, and *Pseudomonas,* found in winter, are all critical in the process of denitrification, which promotes nitrogen cycling in water bodies (Masuda et al., 2017; Yang et al., 2017; He et al., 2019; Zhu et al., 2020).

## 5. Conclusion

This study revealed that the composition of pro- and eukaryotic communities in Lake Dongqian was more specific in different seasons than at different sites, whereas the composition of the pro- and eukaryotic communities at different sites aggregated preferentially in one season. The prokaryotic community was more affected by seasonal changes than the eukaryotic community. TN, pH, Temp and DO significantly affected on both pro- and eukaryotic community compositions, while Chl *a* and NH_4_^+^ only affected on the pro- or eukaryotic community, respectively. The eukaryotic network was more complex than the prokaryotic network, while the number of prokaryotic keystone taxa was larger than that of eukaryotes. The mutual cooperation between pro- and eukaryotic communities across the year was stronger than competition. Most of the microbial keystone taxa involved in N-cycling, such as *Polaromonas*, *Albidiferax*, SM1A02 and *Leptolyngbya,* were significantly correlated with TN, NH_4_^+^, Temp, and Chl *a*, suggesting that they could be used as bio-indicators for the water quality in Lake Dongqian.

## Data availability statement

The datasets presented in this study can be found in online repositories. The names of the repository/repositories and accession number(s) can be found in the article/Supplementary material.

## Author contributions

WH: methodology, data curation, formal analysis, visualization, writing-original draft preparation, and writing-reviewing and editing. SL: investigation, data curation, and formal analysis. SL: data curation and formal analysis. HL: writing-reviewing and editing. QZ: conceptualization, funding acquisition, project administration, resources, investigation, supervision, validation, and writing-reviewing and editing. All authors contributed to the article and approved the submitted version.

## Funding

This study was supported by a grant from Fujian Provincial Department of Science and Technology (2020 N0032), and the National Natural Science Foundation of China (41571252, 32102304, and 41706139), and the Basic Scientific Research Foundation of Zhejiang Provincial Universities.

## Conflict of interest

The authors declare that the research was conducted in the absence of any commercial or financial relationships that could be construed as a potential conflict of interest.

## Publisher’s note

All claims expressed in this article are solely those of the authors and do not necessarily represent those of their affiliated organizations, or those of the publisher, the editors and the reviewers. Any product that may be evaluated in this article, or claim that may be made by its manufacturer, is not guaranteed or endorsed by the publisher.

## Footnotes

^1^ http://www.i-sanger.com/

^2^ http://www.r-project.org
