# Supplementary material for: Pro- and eukaryotic keystone taxa as potential bio-indicators for the water quality of subtropical Lake Dongqian
Source: Front Microbiol. 2023 Apr 26;14:1151768. doi: 10.3389/fmicb.2023.1151768 (PMC10169824; doi:10.3389/fmicb.2023.1151768)
Supplement: Supplementary file 1 [file Data_Sheet_1.docx]

Supplementary Material

Pro- and eukaryotic keystone taxa as potential bio-indicators for the water quality of subtropical Lake Dongqian

Weihong Huang^1^, Shuantong Li^1^, Saisai Li^2^, Hendrikus J. Laanbroek^3,4^, Qiufang Zhang^1 *^

^1^College of Oceanology and Food Science, Quanzhou Normal University, Quanzhou, China

^2^Zhejiang Wanli University, Ningbo, China

^3^Department of Microbial Ecology, Netherlands Institute of Ecology (NIOO-KNAW), Droevendaalsesteeg 10, 6708 PB Wageningen, The Netherlands

^4^Ecology and Biodiversity Group, Department of Biology, Utrecht University, Padualaan 8, 3584 CH Utrecht, The Netherlands

*** Correspondence:** Qiufang Zhang

[qfzhang@1](mailto:r.laanbroek@nioo.knaw.nl)63.com; [qfzhang@qztc.edu.cn](mailto:qfzhang@qztc.edu.cn)

# 1 Supplementary Tables

**Supplementary Table S1.** Geographic information on the water sampling sites at Lake Dongqian.

| **Sites** | **Sampling areas** | **North latitudes** | **East longitudes** |
| --- | --- | --- | --- |
| Site 1 | Northern area | 29°47'00″ | 121° 37'47" |
| Site 2 | Central area | 29°47'18″ | 121° 39'06" |
| Site 3 | Western area | 29°44'26″ | 121° 38'28" |
| Site 4 | Southern area | 29°44'21″ | 121° 39'38" |
| Site 5 | Eastern area | 29°46'11″ | 121° 41'27" |

**Supplementary Table S2.** Conditions applied for Real-time PCR (qPCR) and high-throughput sequencing (HTS) in this study.

| **Target gene** | **Primer** | **Sequence (5′ –3′ )** | **Amplicon length (bp)** | **Thermal profile for qPCR** | **Thermal profile for HTS** | **Reference** |
| --- | --- | --- | --- | --- | --- | --- |
| 16S rRNA | 338F | ACTCCTACGGGAGGCAGCAG | 435 | 2 min at 95℃, followed by 40 cycles of 30 s at 95℃ and 30 s at 55℃. Fluorescence was read during each cycle at 82℃. | 2 min at 95℃, followed by 30 cycles of 30 s at 95℃, 30 s at 55℃ and 30 s at 72℃, and a final extension at 72℃ for 5 min. | Zhang and Laanbroek, 2020 |
|  | 806R | GGACTACHVGGGTWTCTAAT |  |  |  |  |
| 18S rRNA | 817F | TTAGCATGGAATAATRRAATAGGA | 399 | 4 min at 95℃, followed by 40 cycles of 30 s at 95℃ and 30 s at 55℃. Fluorescence was read during each cycle at 82℃. | 3 min at 95℃, followed by 35 cycles of 30 s at 95℃, 30 s at 55℃ and 45 s 72℃, and final extension at 72℃ for 10 min. |  |
|  | 1196R | TCTGGACCTGGTGAGTTTCC |  |  |  |  |

**Supplementary Table S3.** Water properties in Lake Dongqian in spring, summer, autumn and winter.

| **Sampling season** | **Sampling sites** | **Water properties** | | | | | | | | |
| --- | --- | --- | --- | --- | --- | --- | --- | --- | --- | --- |
|  |  | Temp  (℃) | pH | TN  (mg·L^-1^) | NH_4_^+^  (mg·L^-1^) | TP  (mg·L^-1^) | COD_Mn_  (mg·L^-1^) | DO  (mg·L^-1^) | Chl *a*  （mg·m^-3^) | TDS  (g·L^-1^) |
| Spring | Sp1 | 17.5 | 8.09 | 3.34 | 0.43 | 0.09 | 2.65 | 7.98 | 1.59 | 59.1 |
|  | Sp2 | 17.9 | 7.93 | 2.17 | 1.72 | 0.05 | 7.54 | 7.07 | 3.02 | 56.8 |
|  | Sp3 | 18.1 | 7.96 | 3.29 | 0.39 | 0.06 | 3.07 | 8.27 | 1.97 | 57.8 |
|  | Sp4 | 18.8 | 7.94 | 1.47 | 0.90 | 0.09 | 10.99 | 7.03 | 3.37 | 56.4 |
|  | Sp5 | 18.8 | 7.97 | 3.71 | 1.08 | 0.15 | 2.23 | 7.04 | 3.86 | 53.9 |
| Summer | Su1 | 31.2 | 8.43 | 0.57 | 0.17 | 0.06 | 6.74 | 6.25 | 5.58 | 68.0 |
|  | Su2 | 31.9 | 8.24 | 0.52 | 0.17 | 0.12 | 6.23 | 6.20 | 8.37 | 59.0 |
|  | Su3 | 33.3 | 7.63 | 0.50 | 0.16 | 0.12 | 6.32 | 5.80 | 6.28 | 55.6 |
|  | Su4 | 33.1 | 8.04 | 0.83 | 0.17 | 0.14 | 3.64 | 6.55 | 8.37 | 59.9 |
|  | Su5 | 32.5 | 8.44 | 1.01 | 0.15 | 0.14 | 4.15 | 7.30 | 20.46 | 53.5 |
| Autumn | Au1 | 22.9 | 6.65 | 0.23 | 0.10 | 0.13 | 5.62 | 8.66 | 1.86 | 36.6 |
|  | Au2 | 22.6 | 6.38 | 0.45 | 0.10 | 0.11 | 6.63 | 8.71 | 4.65 | 58.1 |
|  | Au3 | 22.9 | 6.43 | 0.28 | 0.09 | 0.09 | 6.11 | 8.64 | 1.86 | 52.0 |
|  | Au4 | 23.0 | 6.55 | 0.46 | 0.09 | 0.10 | 5.79 | 8.65 | 4.65 | 50.2 |
|  | Au5 | 22.8 | 6.19 | 0.30 | 0.22 | 0.16 | 5.59 | 8.70 | 5.58 | 35.5 |
| Winter | Wi1 | 3.7 | 7.99 | 2.20 | 0.22 | 0.19 | 5.55 | 9.83 | 1.22 | 58.2 |
|  | Wi2 | 3.0 | 7.69 | 1.30 | 0.23 | 0.10 | 4.09 | 10.32 | 1.69 | 48.8 |
|  | Wi3 | 5.8 | 7.65 | 2.80 | 0.21 | 0.10 | 3.27 | 10.80 | 1.17 | 85.1 |
|  | Wi4 | 3.0 | 7.79 | 1.30 | 0.22 | 0.45 | 3.16 | 11.46 | 1.86 | 51.7 |
|  | Wi5 | 5.8 | 7.66 | 4.40 | 0.24 | 0.83 | 1.83 | 9.93 | 2.01 | 50.2 |

Note: Sampling sites: Sp1-Sp5, Su1-Su5, Au1-Au5 and Wi1-Wi5 represent five different sampling sites in spring, summer, autumn and winter, respectively. Water properties: Temp = Temperature, TN = total nitrogen, NH_4_^+^ = ammonia, TP = total phosphorus, COD_Mn_ = chemical oxygen demand, DO = dissolved oxygen, Chl *a* = chlorophyll *a*, TDS = total dissolved solids.

**Supplementary Table S4.** The paired comparison of gene abundances and diversity indices between prokaryotes and eukaryotes at different seasons in Lake Dongqian.

| **Microbial index** | **Gene type** | **Sampling season** | | | |
| --- | --- | --- | --- | --- | --- |
|  |  | Spring | Summer | Autumn | Winter |
| Gene abundance (copy number·L-1 water) | 16S | 1.18×10^7^ (1.02×10^7^, 7.30×10^7^) a | 6.35×10^6^ ±  3.64×10^6^ a | 1.04×10^7^ (6.43×10^6^, 1.57×10^7^) b | 8.12×10^6^ (5.36×10^6^, 9.12×10^6^) a |
|  | 18S | 1.03×10^8^ (6.61×10^7^, 4.15×10^8^) a | 5.64×10^6^ ±  4.26×10^6^ a | 9.93×10^8^ (2.87×10^8^, 1.17×10^10^) a | 9.51×10^8^ (5.06×10^6^, 1.42×10^10^) a |
| Shannon | 16S | 4.07 ± 0.67 a | 4.18 ± 0.29 a | 4.56 ± 0.18 a | 4.48 ± 0.17 a |
|  | 18S | 2.63 ± 0.22 b | 3.10 ± 0.28 b | 2.47 ± 0.20 b | 2.03 ± 0.39 b |
| Simpson | 16S | 0.054 ± 0.039 b | 0.049 ± 0.013 b | 0.029 ± 0.007 b | 0.026 ± 0.009 b |
|  | 18S | 0.155 ± 0.028 a | 0.127 ± 0.055 a | 0.222 ± 0.030 a | 0.239 ± 0.056 a |
| Ace | 16S | 719.06 ± 35.92 a | 763.13 ± 26.60 a | 903.31 ± 19.39 a | 738.21 ± 47.13 a |
|  | 18S | 239.83 ± 48.98 b | 286.37 ± 49.99 b | 318.48 ± 13.57b | 197.52 ± 38.24 b |
| Chao | 16S | 742.62 ± 37.55 a | 769.77 ± 26.46 a | 907.33 ± 14.77 a | 758.90 ± 63.20 a |
|  | 18S | 243.99 ± 59.37 b | 292.21 ± 46.39 b | 312.59 ± 13.90 b | 189.28 ± 51.45 b |

Note: Gene type: 16S = 16S rRNA gene, 18S = 18S rRNA gene. Values of five sites in the same season were represented by mean (n=5) ± (SE) when they obeyed normal distribution, and median (IQR) when they disobeyed normal distribution. By paired t-test or Wilcoxon signed rank test, the significant differences in the same index between prokaryotes and eukaryotes in the same season were represented by different letters (*p* <0.05). Due to the absence of a normal distribution, the Wilcoxon signed rank test for Pairing comparison was used for gene abundance between prokaryotes and eukaryotes in Spring, Autumn and Winter.

**Supplementary Table S5.** Percentage distribution **(A)** and taxonomy **(B)** of prokaryotic taxa that discriminate significantly between samples at a log score of at least 4.0 as determined by LDA.

**A**

| **Taxon ID** | **Sampling season** | **Log LDA score** | **Percentage distribution** | | | | | | | | | | | | | | | | | | | |
| --- | --- | --- | --- | --- | --- | --- | --- | --- | --- | --- | --- | --- | --- | --- | --- | --- | --- | --- | --- | --- | --- | --- |
|  |  |  | Sp1 | Sp2 | Sp3 | Sp4 | Sp5 | Su1 | Su2 | Su3 | Su4 | Su5 | Au1 | Au2 | Au3 | Au4 | Au5 | Wi1 | Wi2 | Wi3 | Wi4 | Wi5 |
| **Sp1** | Spring | 4.87 | 46.88 | 25.33 | 3.76 | 5.58 | 0.07 | 0.09 | 0.03 | 0.77 | 0.07 | 2.27 | 0.08 | 0.10 | 0.04 | 0.01 | 0.42 | 1.18 | 10.56 | 4.20 | 2.64 | 0.36 |
| **Sp2** |  | 4.49 | 15.65 | 9.06 | 6.46 | 4.49 | 1.62 | 1.50 | 0.51 | 0.37 | 1.52 | 0.53 | 1.40 | 2.70 | 0.56 | 1.37 | 1.80 | 0.66 | 1.81 | 1.37 | 1.22 | 1.40 |
| **Sp3** |  | 4.48 | 0.55 | 7.47 | 10.79 | 8.73 | 1.28 | 0.03 | 0.13 | 0.09 | 0.21 | 0.06 | 0.14 | 0.11 | 0.22 | 0.25 | 0.16 | 0.16 | 0.13 | 0.16 | 0.04 | 0.22 |
| **Sp4** |  | 4.46 | 15.18 | 8.36 | 5.64 | 4.27 | 1.46 | 1.50 | 0.51 | 0.37 | 1.27 | 0.53 | 1.39 | 2.70 | 0.55 | 1.37 | 1.78 | 0.66 | 1.54 | 1.37 | 1.08 | 1.38 |
| **Sp5** |  | 4.29 | 0.00 | 0.00 | 0.00 | 16.85 | 0.00 | 0.00 | 0.00 | 0.00 | 0.00 | 0.00 | 0.00 | 0.00 | 0.00 | 0.00 | 0.00 | 0.02 | 0.00 | 0.02 | 0.82 | 0.01 |
| **Sp6** |  | 4.01 | 1.04 | 2.23 | 3.52 | 1.57 | 2.66 | 1.20 | 1.32 | 3.34 | 2.15 | 2.16 | 1.45 | 1.64 | 3.58 | 1.23 | 1.04 | 0.28 | 0.08 | 0.10 | 0.08 | 0.55 |
| **Su^1^** | Summer | 5.07 | 2.03 | 1.91 | 2.36 | 1.05 | 13.79 | 33.32 | 29.73 | 26.07 | 18.12 | 28.25 | 6.21 | 7.07 | 8.2 | 11.17 | 4.61 | 13.88 | 0.79 | 7.67 | 1.27 | 3.43 |
| **Su^2^** |  | 4.07 | 0.05 | 0.02 | 0.03 | 0.01 | 1.16 | 1.53 | 1.16 | 1.40 | 4.14 | 3.93 | 0.58 | 1.03 | 2.12 | 3.68 | 0.33 | 0.09 | 0.00 | 0.03 | 0.01 | 0.02 |
| **Su^3^** |  | 4.06 | 0.01 | 0.01 | 0.03 | 0.01 | 0.02 | 2.78 | 1.87 | 4.32 | 1.46 | 1.36 | 0.71 | 1.24 | 2.34 | 0.77 | 0.85 | 0.01 | 0.00 | 0.02 | 0.01 | 0.04 |
| **Au^1^** | Autumn | 4.84 | 1.13 | 7.9 | 11.58 | 11.14 | 4.00 | 7.86 | 4.57 | 7.63 | 6.64 | 3.73 | 17.68 | 12.53 | 17.88 | 10.69 | 14.15 | 0.57 | 0.45 | 0.48 | 0.18 | 0.76 |
| **Au^2^** |  | 4.79 | 4.07 | 7.45 | 7.82 | 6.25 | 6.92 | 19.19 | 24.08 | 19.97 | 14.91 | 8.11 | 24.80 | 16.76 | 19.89 | 15.94 | 20.68 | 14.89 | 2.24 | 14.57 | 3.49 | 12.25 |
| **Au^3^** |  | 4.51 | 0.45 | 5.53 | 6.03 | 4.64 | 2.54 | 0.33 | 0.69 | 1.10 | 0.59 | 0.61 | 3.33 | 7.50 | 9.12 | 5.57 | 6.07 | 0.11 | 0.12 | 0.09 | 0.21 | 0.35 |
| **Au^4^** |  | 4.36 | 1.16 | 3.24 | 4.23 | 3.51 | 3.80 | 2.96 | 3.30 | 4.05 | 6.36 | 1.98 | 4.89 | 3.03 | 6.98 | 6.97 | 4.46 | 1.24 | 0.49 | 0.95 | 0.24 | 1.73 |
| **Au^5^** |  | 4.26 | 0.02 | 0.10 | 0.19 | 0.06 | 0.34 | 0.05 | 0.01 | 0.99 | 0.10 | 0.05 | 4.70 | 6.04 | 1.01 | 1.67 | 8.06 | 0.02 | 0.02 | 0.11 | 0.53 | 0.06 |
| **Au^6^** |  | 4.01 | 1.16 | 1.25 | 1.08 | 0.45 | 0.34 | 2.59 | 1.30 | 0.94 | 3.85 | 1.32 | 2.42 | 2.60 | 2.21 | 3.04 | 2.89 | 0.30 | 0.26 | 0.46 | 0.57 | 1.53 |
| **Wi^1^** | Winter | 4.41 | 0.05 | 0.06 | 0.09 | 0.24 | 1.15 | 0.15 | 0.03 | 0.09 | 0.04 | 0.68 | 0.00 | 0.09 | 0.00 | 0.00 | 0.04 | 1.52 | 7.00 | 3.07 | 10.48 | 5.53 |
| **Wi^2^** |  | 4.37 | 0.01 | 0.00 | 0.00 | 0.00 | 0.01 | 0.00 | 0.00 | 0.00 | 0.00 | 0.00 | 0.00 | 0.00 | 0.00 | 0.00 | 0.00 | 0.23 | 17.53 | 1.24 | 2.52 | 0.35 |
| **Wi^3^** |  | 4.36 | 0.10 | 0.13 | 0.49 | 0.07 | 0.77 | 0.01 | 0.00 | 0.00 | 0.00 | 0.01 | 0.00 | 0.00 | 0.00 | 0.00 | 0.00 | 4.18 | 0.34 | 7.63 | 5.09 | 6.31 |
| **Wi^4^** |  | 4.35 | 1.19 | 4.77 | 5.54 | 1.90 | 3.95 | 0.78 | 0.99 | 0.43 | 0.71 | 0.69 | 1.31 | 1.98 | 0.99 | 1.64 | 1.61 | 8.03 | 2.60 | 5.10 | 3.01 | 7.15 |
| **Wi^5^** |  | 4.30 | 0.72 | 0.89 | 1.52 | 0.33 | 2.45 | 0.05 | 0.01 | 0.05 | 0.03 | 0.21 | 0.01 | 0.80 | 0.03 | 0.00 | 0.14 | 2.52 | 0.21 | 4.38 | 9.41 | 6.08 |
| **Wi^6^** |  | 4.25 | 0.25 | 0.39 | 0.32 | 0.23 | 0.07 | 0.00 | 0.00 | 0.00 | 0.00 | 0.00 | 0.00 | 0.01 | 0.00 | 0.00 | 0.00 | 2.67 | 4.29 | 2.88 | 6.47 | 1.87 |
| **Wi^7^** |  | 4.24 | 0.07 | 0.23 | 0.30 | 0.20 | 0.16 | 0.04 | 0.01 | 0.04 | 0.08 | 0.06 | 0.02 | 0.20 | 0.09 | 0.12 | 0.05 | 2.66 | 0.40 | 4.70 | 3.43 | 7.39 |
| **Wi^8^** |  | 4.22 | 0.31 | 0.62 | 0.98 | 0.21 | 1.69 | 1.58 | 0.81 | 0.83 | 1.34 | 0.89 | 0.92 | 2.99 | 0.85 | 0.71 | 0.79 | 2.81 | 1.21 | 4.04 | 7.22 | 6.92 |
| **Wi^9^** |  | 4.21 | 0.00 | 0.02 | 0.19 | 0.02 | 0.03 | 0.00 | 0.00 | 0.00 | 0.01 | 0.00 | 0.00 | 0.00 | 0.00 | 0.00 | 0.00 | 0.10 | 12.26 | 0.44 | 1.81 | 0.93 |
| **Wi^10^** |  | 4.03 | 0.04 | 0.08 | 0.29 | 0.01 | 0.04 | 0.03 | 0.02 | 0.01 | 0.01 | 0.07 | 0.01 | 0.02 | 0.00 | 0.00 | 0.01 | 0.70 | 4.40 | 3.55 | 0.77 | 0.30 |
| **Wi^11^** |  | 4.02 | 0.87 | 1.78 | 1.77 | 0.49 | 1.36 | 0.86 | 0.56 | 0.15 | 0.34 | 0.66 | 0.16 | 0.58 | 0.28 | 0.44 | 0.47 | 2.64 | 0.25 | 3.14 | 3.95 | 3.13 |

**B**

| **Taxon ID** | **Taxonomy** | | | | |
| --- | --- | --- | --- | --- | --- |
|  | Phylum | Class | Order | Family | Genus |
| Sp1 | Proteobacteria | Gammaproteobacteria | Pseudomonadales |  |  |
| Sp2 | Actinobacteria | Actinobacteria | Corynebacteriales |  |  |
| Sp3 | Cyanobacteria | Cyanobacteria | Subsection I | Family I of Subsection I | *Microcystis* |
| Sp4 | Actinobacteria | Actinobacteria | Corynebacteriales | Mycobacteriaceae | *Mycobacterium* |
| Sp5 | Proteobacteria | Epsilonproteobacteria | Campylobacterales |  |  |
| Sp6 | Verrucomicrobia | Spartobacteria | Chthoniobacterales |  |  |
| Su^1^ | Actinobacteria | Actinobacteria | Frankiales | Sporichthyaceae | hgcI clade |
| Su^2^ | Verrucomicrobia | Verrucomicrobia Incertae Sedis | Unknown | Unknown | *Candidatus* Methylacidiphilum |
| Su^3^ | Proteobacteria | Betaproteobacteria | Burkholderiales | Comamonadaceae | *Sphaerotilus* |
| Au ^1^ | Cyanobacteria | Cyanobacteria | Subsection I | Family I Subsection I | *Synechococcus* |
| Au ^2^ | Actinobacteria | Actinobacteria | Acidimicrobiales | Acidimicrobiaceae | CL500-29 marine group |
| Au ^3^ | Saccharibacteria | No rank | No rank | No rank | No rank |
| Au ^4^ | Cyanobacteria | Cyanobacteria | Subsection I | Family I of Subsection I | Unclassified |
| Au^5^ | Firmicutes | Bacilli | Bacillales | Bacillaceae | *Bacillus* |
| Au^6^ | Actinobacteria | Actinobacteria | PeM15 | No rank | No rank |
| Wi^1^ | Bacteroidetes | Flavobacteriia | Flavobacteriales | Flavobacteriaceae | *Flavobacterium* |
| Wi^2^ | Proteobacteria | Betaproteobacteria | Burkholderiales | Oxalobacteraceae | *Massilia* |
| Wi^3^ | Proteobacteria | Betaproteobacteria | Burkholderiales | Comamonadaceae | *Rhodoferax* |
| Wi^4^ | Cyanobacteria | Cyanobacteria | No rank | No rank | No rank |
| Wi^5^ | Proteobacteria | Betaproteobacteria | Burkholderiales | Comamonadaceae | *Limnohabitans* |
| Wi^6^ | Proteobacteria | Betaproteobacteria | Burkholderiales | Alcaligenaceae | GKS98 freshwater group |
| Wi^7^ | Proteobacteria | Alphaproteobacteria | Rhodobacterales | Rhodobacteraceae | *Rhodobacter* |
| Wi^8^ | Proteobacteria | Betaproteobacteria | Burkholderiales | Comamonadaceae | Unclassified |
| Wi^9^ | Actinobacteria | Actinobacteria | Micrococcales | Micrococcaceae | Unclassified |
| Wi^10^ | Proteobacteria | Gammaproteobacteria | Pseudomonadales | Pseudomonadaceae | *Pseudomonas* |
| Wi^11^ | Proteobacteria | Betaproteobacteria | Burkholderiales | Burkholderiaceae | *Polynucleobacter* |

Note: Taxon ID: Sp^1^-Sp^6^, Su^1^-Su^3^, Au^1^-Au^6^ and Wi^1^-Wi^11^ represent the distinctive prokaryotic taxa in Spring, Summer, Autumn, and Winter, respectively; Sp1-Sp5, Su1-Su5, Au1-Au5 and Wi1-Wi5 represent samples collected at the different sampling sites in spring, summer, autumn and winter.

**Supplementary Table S6.** Percentage distribution (A) and taxonomy (B) of eukaryotic taxa that discriminate significantly between samples at a log score of at least 4.0 as determined by LDA.

**A**

| **Taxon ID** | **Sampling season** | **Log LDA score** | **Percentage distribution** | | | | | | | | | | | | | | | | | | | |
| --- | --- | --- | --- | --- | --- | --- | --- | --- | --- | --- | --- | --- | --- | --- | --- | --- | --- | --- | --- | --- | --- | --- |
|  |  |  | Sp1 | Sp2 | Sp3 | Sp4 | Sp5 | Su1 | Su2 | Su3 | Su4 | Su5 | Au1 | Au2 | Au3 | Au4 | Au5 | Wi1 | Wi2 | Wi3 | Wi4 | Wi5 |
| **Sp1** | Spring | 4.91 | **3.77** | **29.11** | **7.88** | **29.17** | **12.20** | 0.01 | 0.00 | 0.00 | 0.00 | 0.01 | 0.04 | 0.10 | 0.07 | 0.00 | 0.89 | 2.05 | 3.00 | 1.71 | 1.42 | 9.44 |
| **Sp2** |  | 4.65 | **23.90** | **9.36** | **9.02** | **3.28** | **2.35** | 2.06 | 4.56 | 12.79 | 1.61 | 1.74 | 0.78 | 2.39 | 1.98 | 3.71 | 0.40 | 0.00 | 0.30 | 0.00 | 0.03 | 0.02 |
| **Sp3** |  | 4.63 | **15.01** | **13.61** | **7.74** | **14.48** | **3.17** | 6.63 | 6.94 | 0.90 | 1.82 | 1.23 | 4.82 | 10.43 | 8.11 | 9.65 | 12.75 | 3.30 | 1.68 | 1.78 | 1.16 | 0.56 |
| **Sp4** |  | 4.06 | **1.02** | **2.25** | **1.24** | **2.44** | **4.41** | 0.39 | 0.18 | 0.16 | 0.10 | 0.05 | 0.42 | 0.32 | 0.17 | 0.03 | 0.28 | 1.93 | 1.53 | 1.36 | 0.71 | 3.27 |
| **Su^1^** | Summer | 5.22 | 0.13 | 0.30 | 0.46 | 0.54 | 0.62 | **44.86** | **35.80** | **50.57** | **16.18** | **13.60** | 14.04 | 7.00 | 6.16 | 13.36 | 5.21 | 0.04 | 1.42 | 0.14 | 1.29 | 0.97 |
| **Su^2^** |  | 4.59 | 0.01 | 0.09 | 0.02 | 0.00 | 0.01 | **2.50** | **5.74** | **4.47** | **13.14** | **14.84** | 0.63 | 0.99 | 0.73 | 0.89 | 0.31 | 0.00 | 0.00 | 0.00 | 0.00 | 0.00 |
| **Su^3^** |  | 4.40 | 2.16 | 5.92 | 2.73 | 3.83 | 4.64 | **5.43** | **6.12** | **4.74** | **8.08** | **6.12** | 2.32 | 1.89 | 2.21 | 3.72 | 1.45 | 0.63 | 1.30 | 0.80 | 0.84 | 2.25 |
| **Su^4^** |  | 4.03 | 0.02 | 0.07 | 0.12 | 0.36 | 0.13 | **1.55** | **5.13** | **1.36** | **1.68** | **0.85** | 0.07 | 0.21 | 0.25 | 0.34 | 0.08 | 0.03 | 0.05 | 0.04 | 0.03 | 0.01 |
| **Au^1^** | Autumn | 5.34 | 1.27 | 1.84 | 0.52 | 0.58 | 0.42 | 10.71 | 6.74 | 1.41 | 3.21 | 10.68 | **48.17** | **43.39** | **40.41** | **43.27** | **48.82** | 46.89 | 31.02 | 45.65 | 37.94 | 7.04 |
| **Au^2^** |  | 4.70 | 2.45 | 8.57 | 15.50 | 4.42 | 7.49 | 1.30 | 0.50 | 0.31 | 1.15 | 0.65 | **16.66** | **1.27** | **17.28** | **0.90** | **12.47** | 3.18 | 10.94 | 6.02 | 4.33 | 7.56 |
| **Au^3^** |  | 4.04 | 0.87 | 2.98 | 1.73 | 1.38 | 1.49 | 0.15 | 0.06 | 0.07 | 0.53 | 1.62 | **2.14** | **3.82** | **3.46** | **0.66** | **2.85** | 0.22 | 0.51 | 0.50 | 0.41 | 1.58 |
| **Wi^1^** | Winter | 5.20 | 36.62 | 7.27 | 5.68 | 27.87 | 31.52 | 3.49 | 6.31 | 0.11 | 0.50 | 0.62 | 2.29 | 14.04 | 4.00 | 4.06 | 6.44 | **31.37** | **20.72** | **24.59** | **36.53** | **58.51** |
| **Wi^2^** |  | 4.37 | 2.62 | 1.61 | 1.24 | 4.05 | 5.76 | 1.14 | 0.29 | 0.00 | 0.11 | 0.22 | 0.31 | 1.12 | 0.45 | 0.45 | 0.78 | **4.53** | **2.83** | **4.28** | **4.40** | **5.35** |

**B**

| **Taxon ID** | **Taxonomy** | | | | |
| --- | --- | --- | --- | --- | --- |
|  | Phylum | Class | Order | Family | Genus |
| Sp1 | Kathablepharidae | No rank | No rank | No rank | No rank |
| Sp2 | Cryptomycota | No rank | No rank | No rank | No rank |
| Sp3 | Ciliophora | Intramacronucleata | Spirotrichea | Choreotrichia | *Tintinnidium* |
| Sp4 | Ciliophora | Intramacronucleata | Conthreep | Prostomatea | *Cryptocaryon* |
| Su^1^ | Chytridiomycota | No rank | No rank | No rank | No rank |
| Su^2^ | Choanoflagellida | Craspedida | Salpingoecidae | No rank | *Salpingoeca* |
| Su^3^ | Unclassified_d__Eukaryota | Unclassified | Unclassified | Unclassified | Unclassified |
| Su^4^ | Unclassified_k__Fungi | Unclassified | Unclassified | Unclassified | Unclassified |
| Au^1^ | Cryptomonadales | No rank | No rank | No rank | *Cryptomonas* |
| Au^2^ | Cryptomonadales | No rank | No rank | No rank | No rank |
| Au^3^ | Cryptomonadales | No rank | No rank | No rank | Unclassified |
| Wi^1^ | Ciliophora | Intramacronucleata | Spirotrichea | Choreotrichia | No rank |
| Wi^2^ | Ciliophora | Intramacronucleata | Spirotrichea | Choreotrichia | Unclassified |

Note: Taxon ID: Sp^1^-Sp^4^, Su^1^-Su^4^, Au^1^-Au^3^ and Wi^1^-Wi^2^ represent the distinctive eukaryotic taxa in Spring, Summer, Autumn, and Winter, respectively; Sp1-Sp5, Su1-Su5, Au1-Au5 and Wi1-Wi5 represent samples collected at the different sampling sites in spring, summer, autumn and winter

# 2 Supplementary Figures


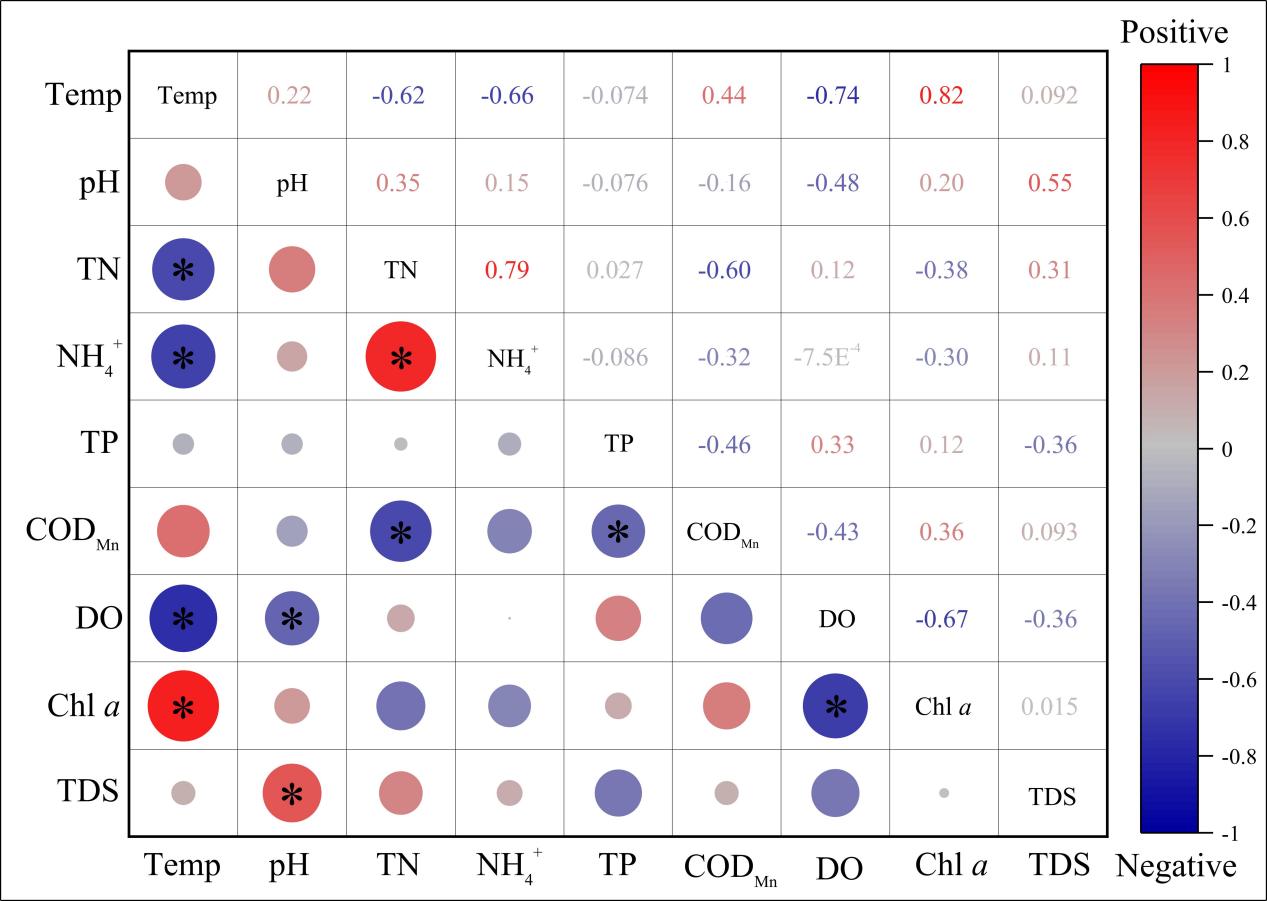


**Supplementary Figure 1.** Spearman correlation heatmaps for the relationships between water properties. The R value is displayed in different colors (*: *p* < 0.05).

**
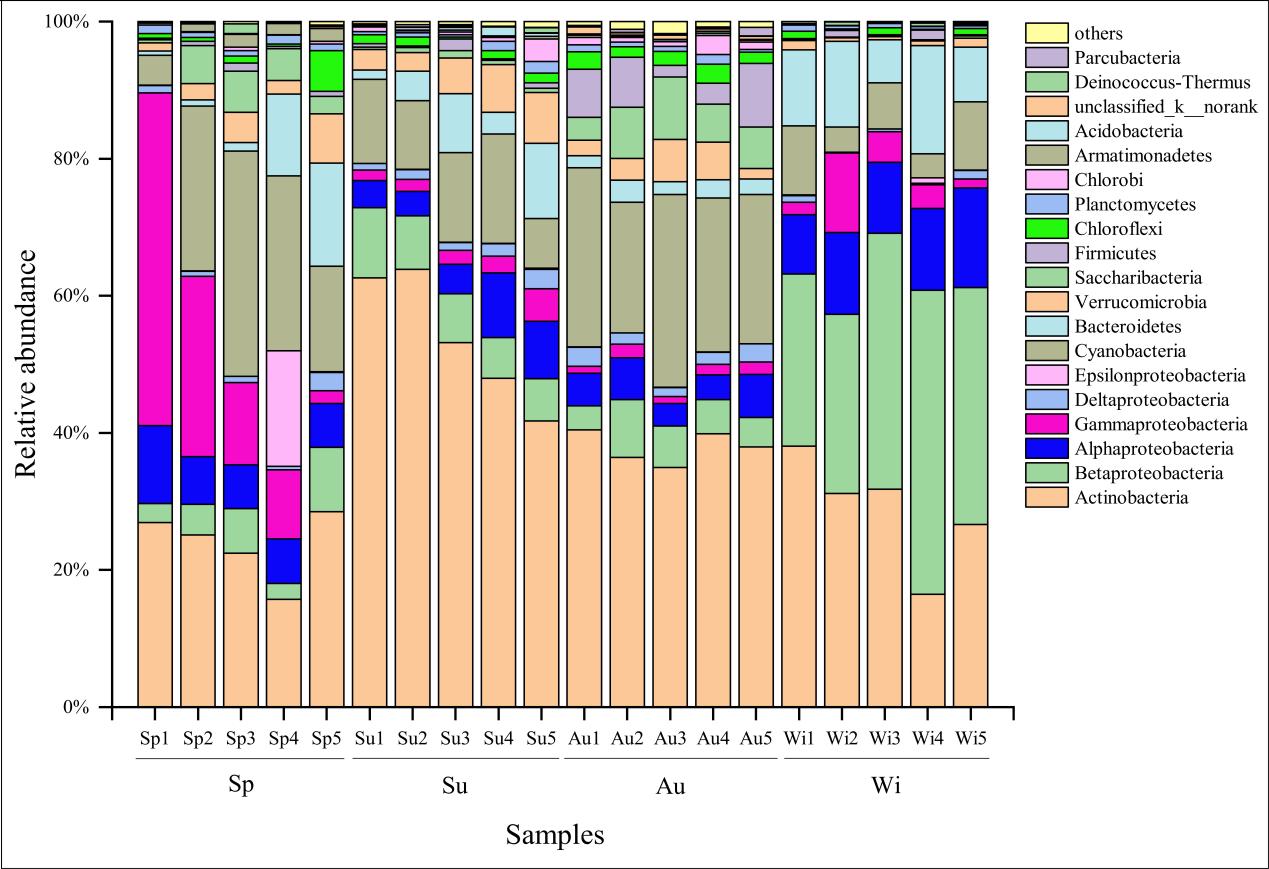
**

**Supplementary Figure 2.** Prokaryotic community composition in different seasons and sampling sites in Lake Dongqian as revealed by high-throughput sequencing (HTS) analysis. Presented are taxa that exceeded 1% relative abundance at the phylum level, except for members of the phylum Proteobacteria, which are shown at the class level. Sp1-Sp5, Su1-Su5, Au1-Au5 and Wi-Wi5 represent different samples collected at the sampling sites in spring, summer, autumn and winter, respectively.


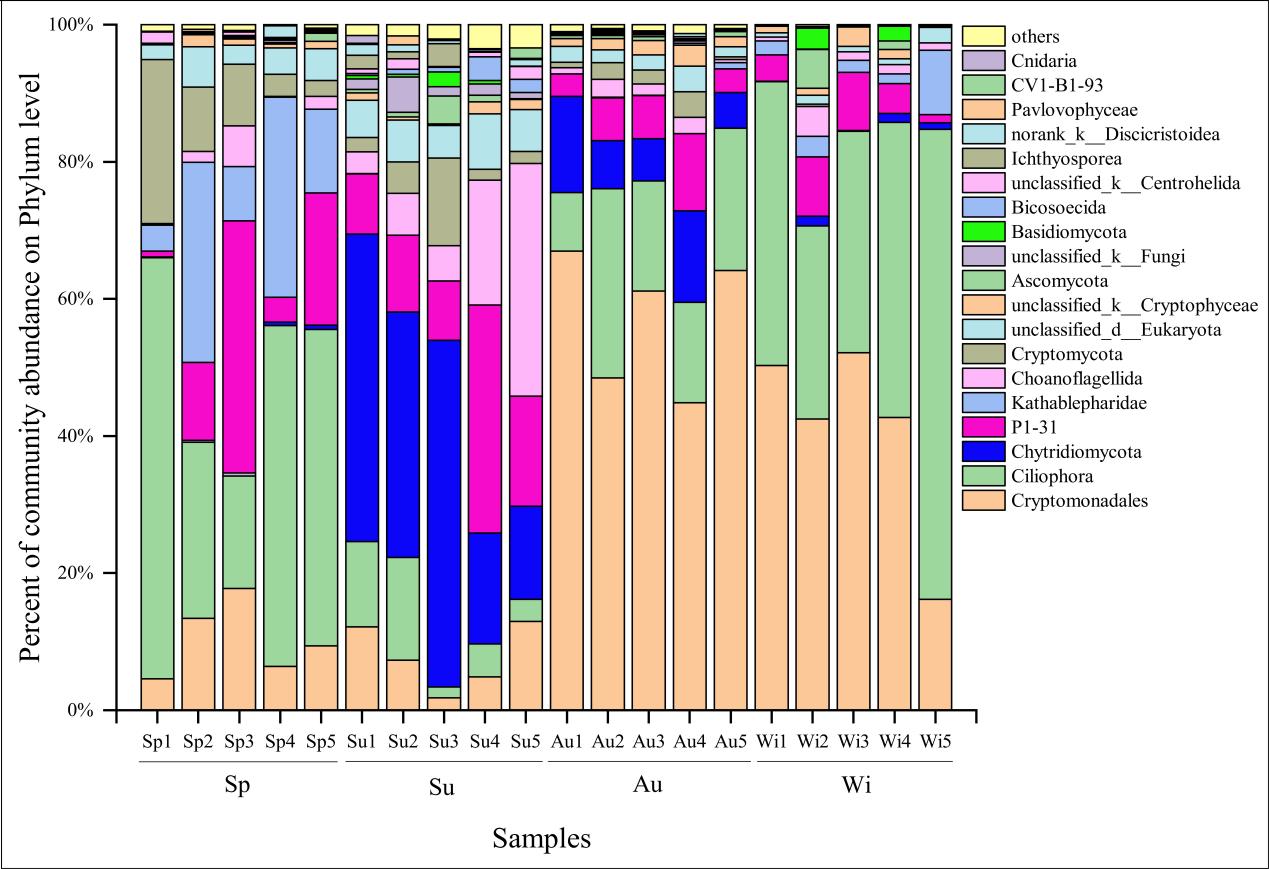


**Supplementary Figure S3.** Eukaryotic community composition in the different seasons and sampling sites in Lake Dongqian as revealed by high-throughput sequencing (HTS) analysis. Presented are taxa that exceeded 1% relative abundance at the phylum level. Sp1-Sp5, Su1-Su5, Au1-Au5 and Wi1-Wi5 represent samples collected at different sampling sites in spring, summer, autumn and winter.

**A**

**
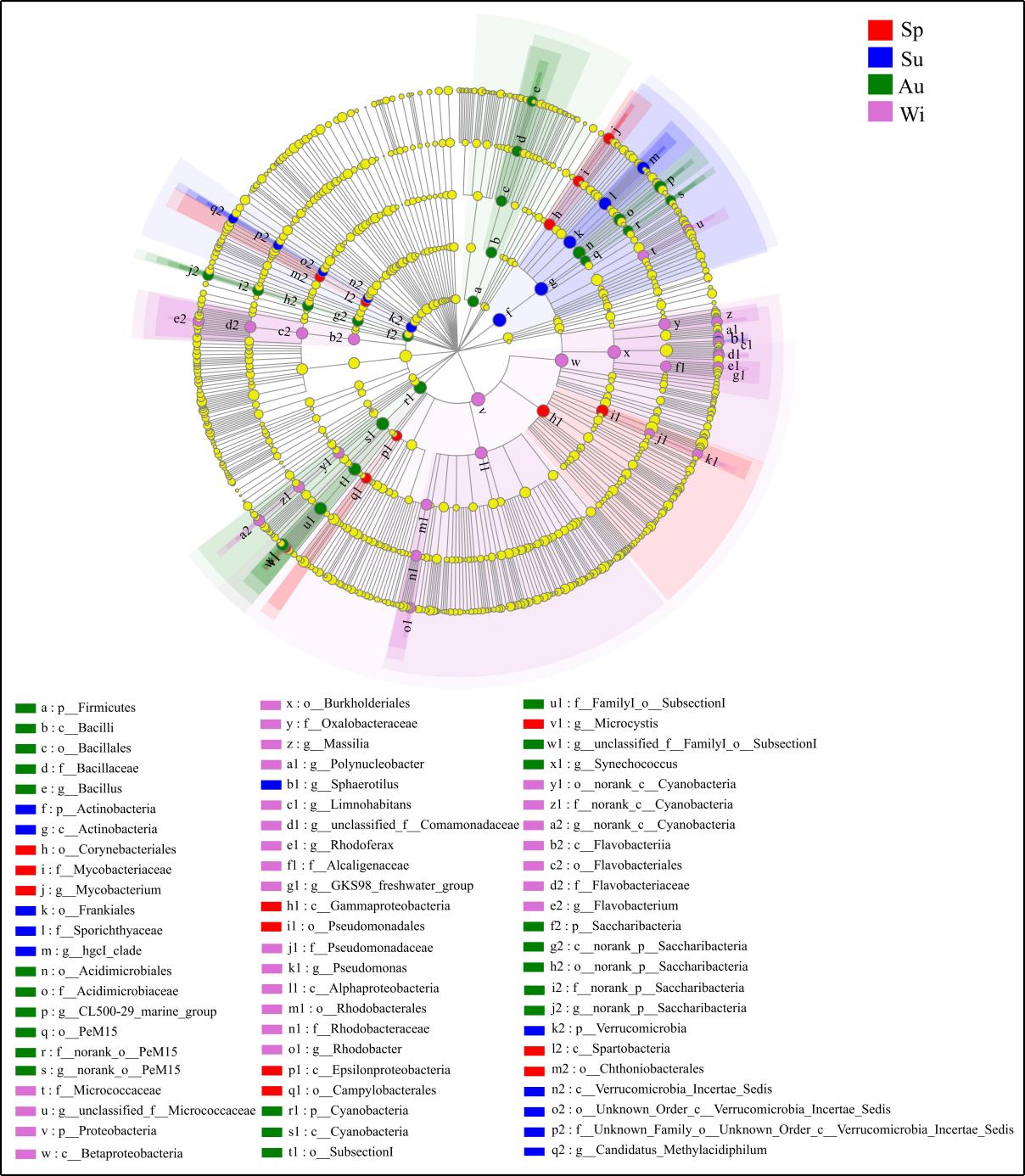
**

**B**

**
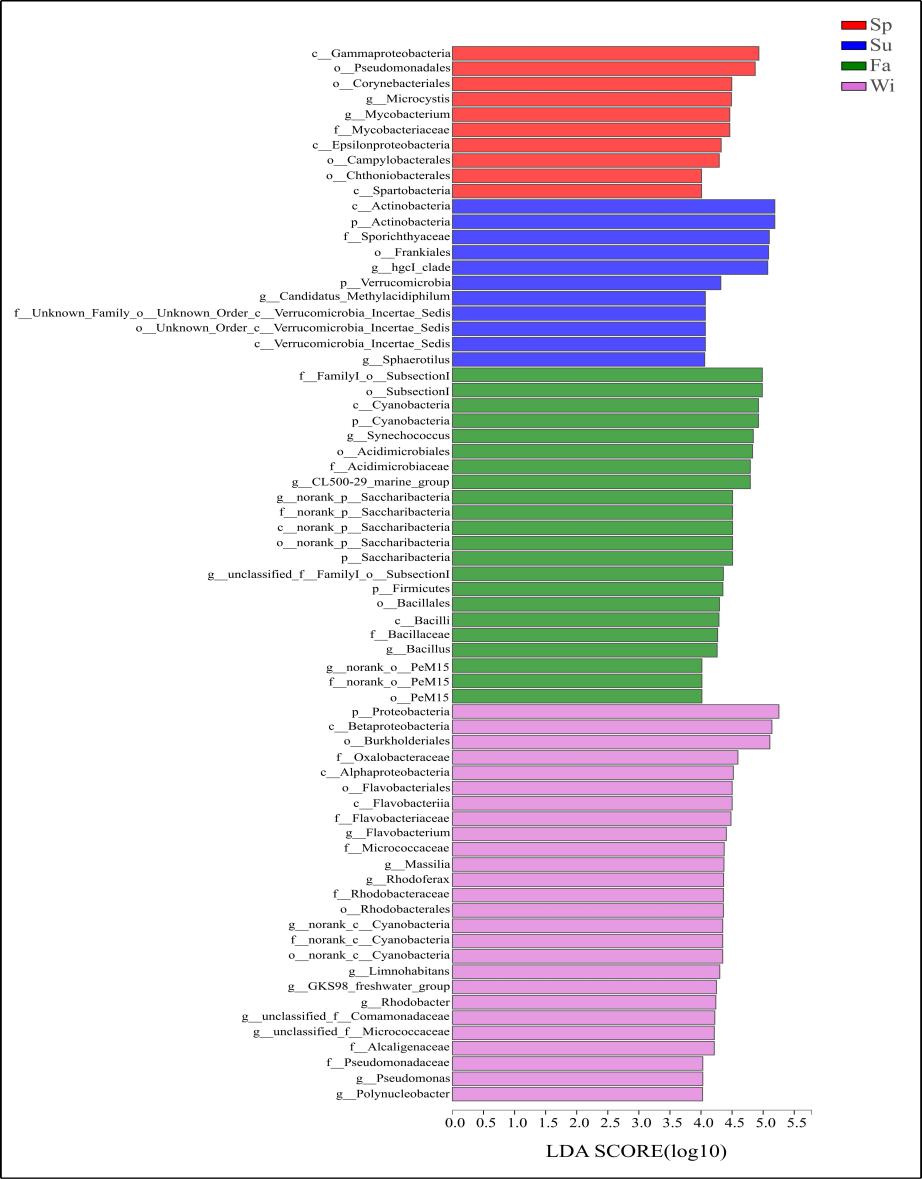
**

**Supplementary Figure S4.** Cladogram showing the phylogenetic distribution of prokaryotic lineages with the four seasons **(A)**. Indicator bacteria with LDA scores of 4.0 or greater at each site are also listed **(B)**. Sp, Su, Au, and Wi represent spring, summer, autumn and winter, respectively. In the legend, phylum, class, order, family and genus levels are indicated by p, c, o, f and g, respectively, before the names of the taxa.

**A**

**
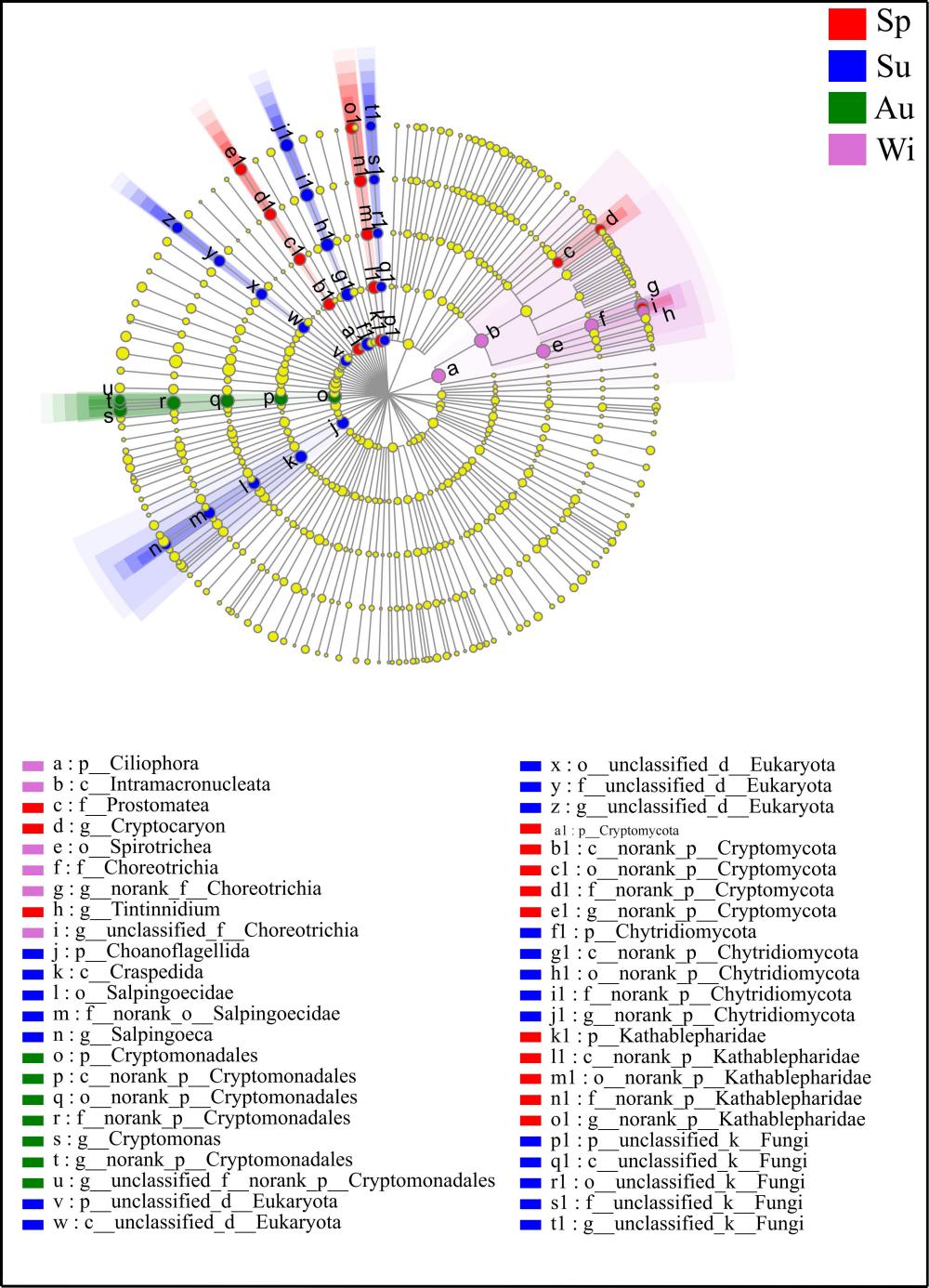
**

**B**

**
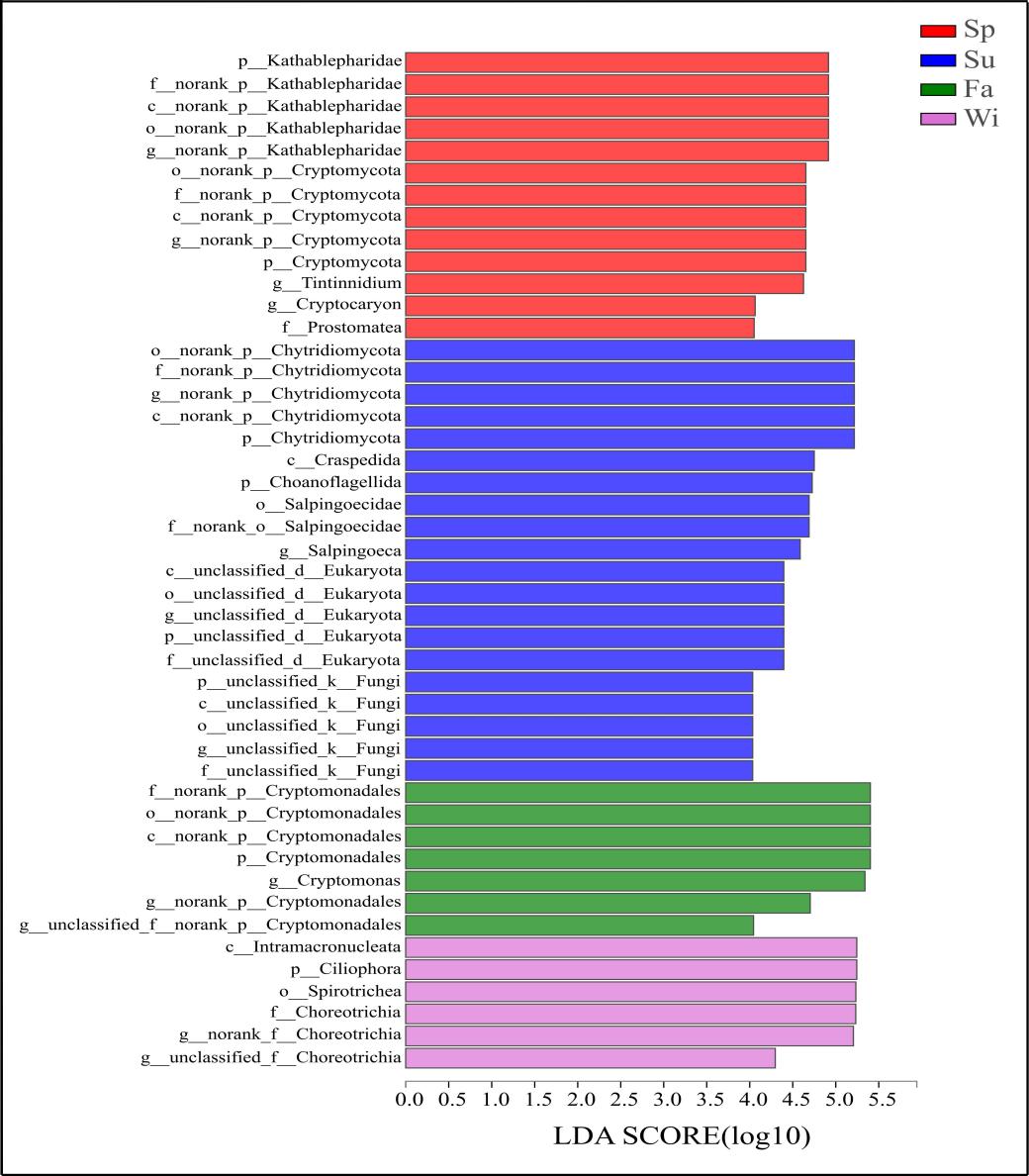
**

**Supplementary Figure S5.** Cladogram showing the phylogenetic distribution of eukaryotic lineages with the four seasons **(A)**. Indicator bacteria with LDA scores of 4.0 or greater at each site are also listed **(B)**. Sp, Su, Au, and Wi represent spring, summer, autumn and winter, respectively. In the legend, phylum, class, order, family and genus levels are indicated by p, c, o, f and g, respectively, before the names of the taxa.


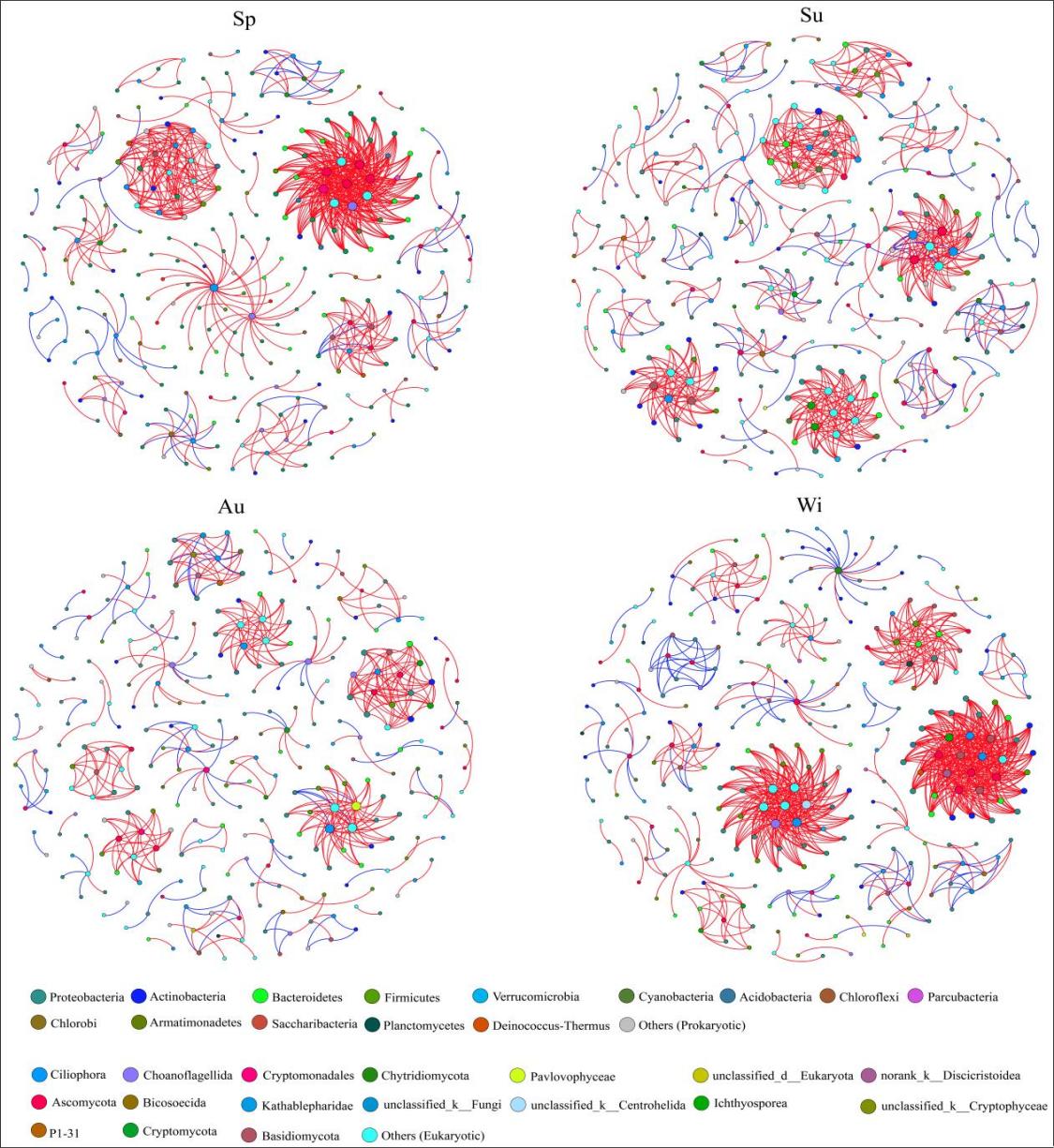


**Supplementary Figure S6.** Correlation network graphs for the relationship between prokaryotic and eukaryotic taxa at the genus level in Lake Dongqian in the different seasons. Sp, Su, Au and Wi represent spring, summer, autumn and winter, respectively. Each node signifies a genus, colors of the nodes indicate different phyla, while phyla with relative abundance < 1% are merged as “others”. The size of each node is proportional to the number of edges (*i.e*., degree). A red edge indicates a significant positive correlation (*p* < 0.05) between two individual nodes, while a blue edge indicates a significant negative correlation (*p* < 0.05).

**A**

**
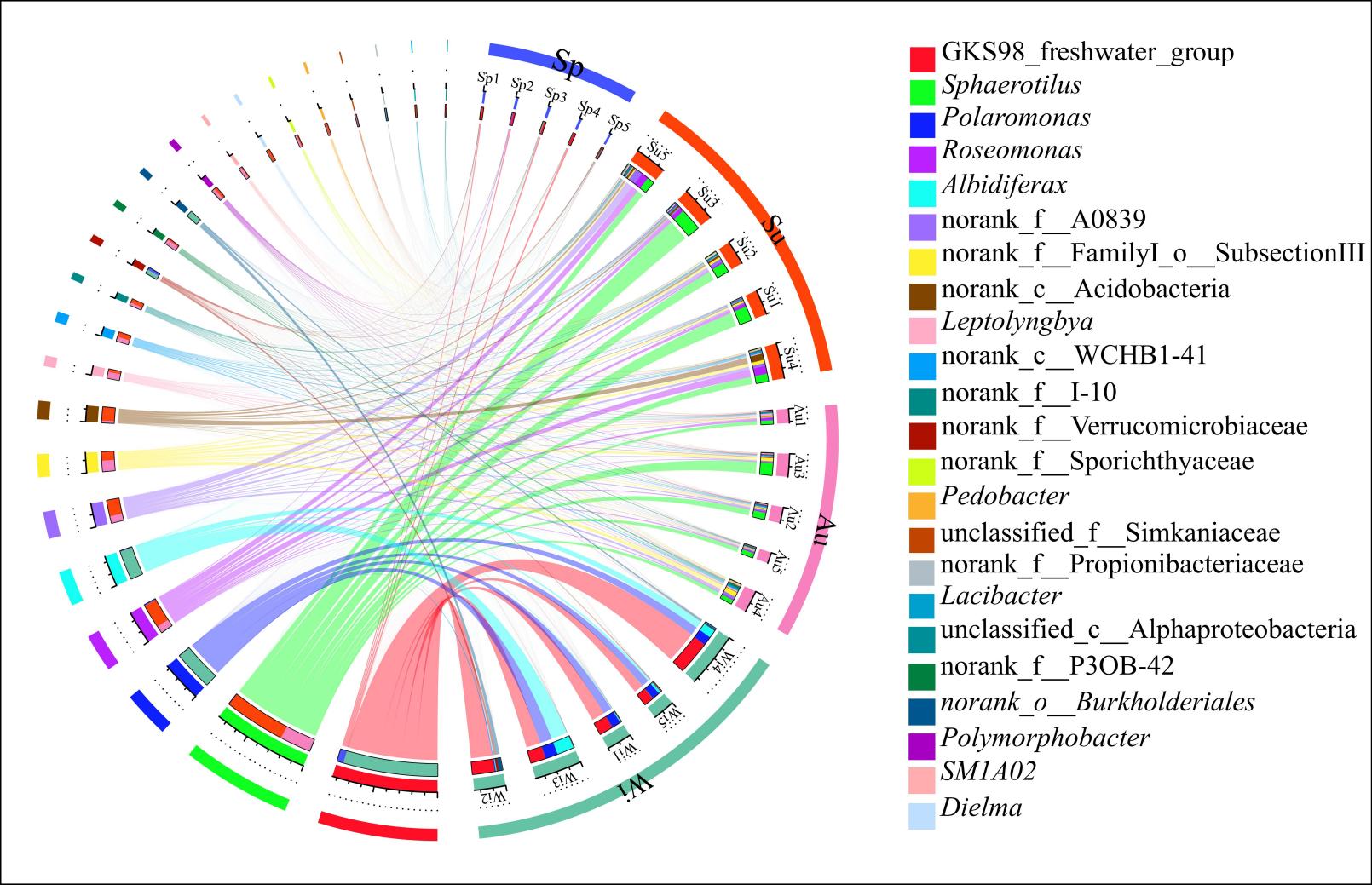
**

**B**

**
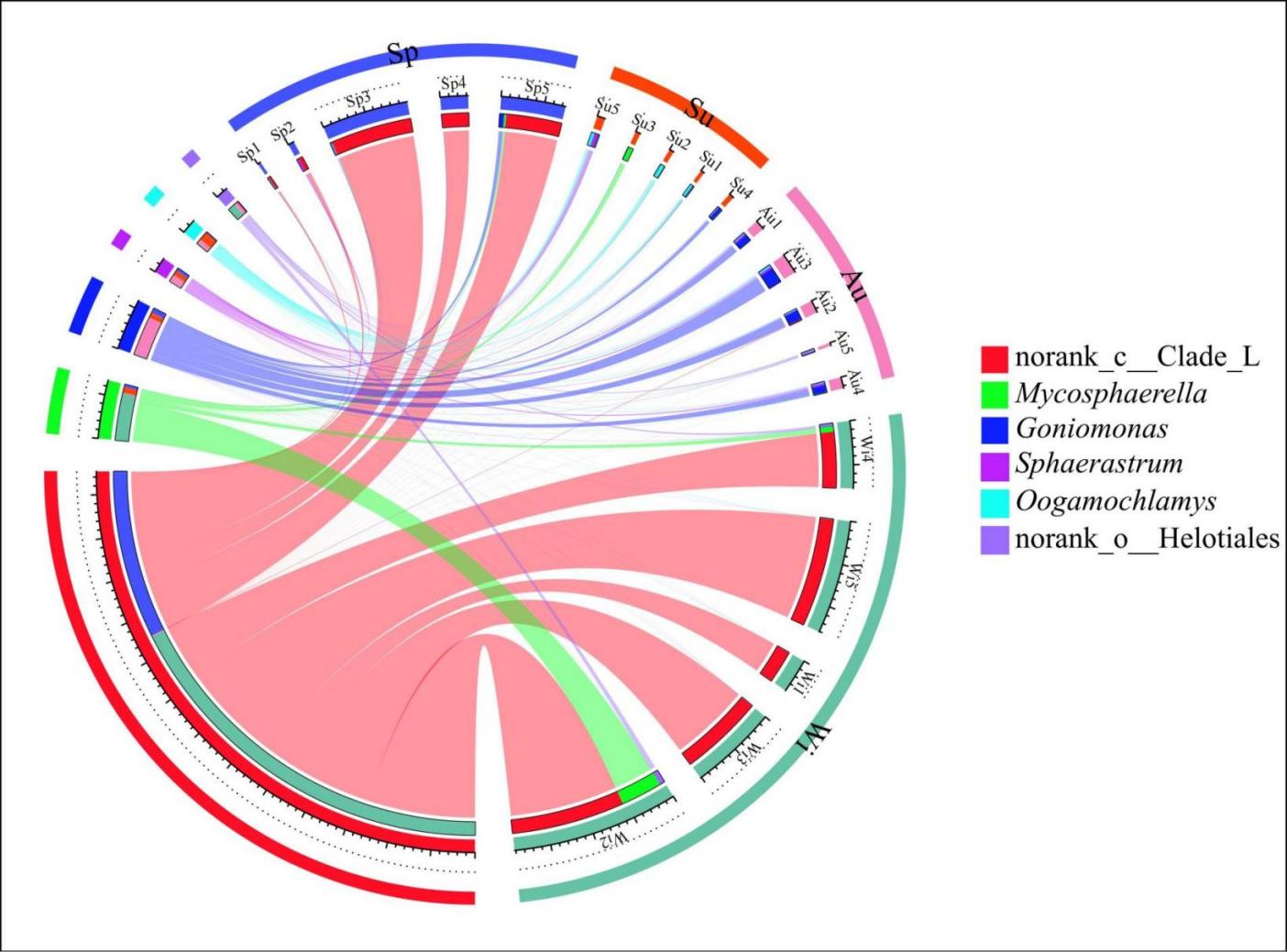
**

**Supplementary Figure S7.** Chord diagram of the relationships between keystone taxa in the prokaryotic **(A)** and eukaryotic **(B)** co-occurrence networks in relation to seasons. Sp, Su, Au, and Wi represent spring, summer, autumn and winter, respectively, and Sp1-Sp5, Su1-Su5, Au1-Au5 and Wi1-Wi5 represent the samples collected at the different sampling sites in spring, summer, autumn and winter.
